# ERG activity is regulated by endothelial FAK coupling with TRIM25/USP9x in vascular patterning

**DOI:** 10.1242/dev.200528

**Published:** 2022-07-12

**Authors:** Gabriela D'Amico, Isabelle Fernandez, Jesús Gómez-Escudero, Hyojin Kim, Eleni Maniati, Muhammad Syahmi Azman, Faraz K. Mardakheh, Bryan Serrels, Alan Serrels, Maddy Parsons, Anthony Squire, Graeme M. Birdsey, Anna M. Randi, Alfonso Bolado-Carrancio, Rathi Gangeswaran, Louise E. Reynolds, Natalia Bodrug, Yaohe Wang, Jun Wang, Pascal Meier, Kairbaan M. Hodivala-Dilke

**Affiliations:** 1 Centre for Tumour Microenvironment, Barts Cancer Institute – a CR-UK Centre of Excellence, Queen Mary University of London, John Vane Science Centre, Charterhouse Square, London EC1M 6BQ, UK; 2 The Breakthrough Toby Robins Breast Cancer Research Centre, Institute of Cancer Research, Chester Beatty Laboratories, Fulham Road, London SW3 6JB, UK; 3 Centre for Cancer Genomics and Computational Biology, Barts Cancer Institute – a CR-UK Centre of Excellence, Queen Mary University of London, John Vane Science Centre, Charterhouse Square, London EC1M 6BQ, UK; 4 Centre for Cancer Cell and Molecular Biology, Barts Cancer Institute – a CR-UK Centre of Excellence, Queen Mary University of London, John Vane Science Centre, Charterhouse Square, London EC1M 6BQ, UK; 5 Institute of Cancer Sciences, University of Glasgow, Garscube Estate, Switchback Road, Bearsden G61 1QH, UK; 6 Centre for Inflammation Research, Queen's Medical Research Institute, University of Edinburgh, Edinburgh BioQuarter, 47 Little France Crescent, Edinburgh EH16 4TJ, UK; 7 Kings College London, Randall Centre of Cell and Molecular Biophysics, Room 3.22B, New Hunts House, Guys Campus, London SE1 1UL, UK; 8 IMCES - Imaging Centre Essen, Institute for Experimental Immunology and Imaging, University Clinic Essen, Hufelandstrasse 55, 45122 Essen, Germany; 9 National Heart & Lung Institute, Imperial College London, Hammersmith Hospital, Du Cane Road, London W12 0NN, UK; 10 Institute of Genetics and Cancer, University of Edinburgh, Edinburgh EH4 2XU, UK; 11 Centre for Cancer Biomarkers and Biotherapeutics, Barts Cancer Institute – a CR-UK Centre of Excellence, Queen Mary University of London, John Vane Science Centre, Charterhouse Square, London EC1M 6BQ, UK

**Keywords:** Vascular patterning regulation, Retinal angiogenesis, Endothelial cell signalling

## Abstract

Precise vascular patterning is crucial for normal growth and development. The ERG transcription factor drives Delta-like ligand 4 (DLL4)/Notch signalling and is thought to act as a pivotal regulator of endothelial cell (EC) dynamics and developmental angiogenesis. However, molecular regulation of ERG activity remains obscure. Using a series of EC-specific focal adhesion kinase (FAK)-knockout (KO) and point-mutant FAK-knock-in mice, we show that loss of ECFAK, its kinase activity or phosphorylation at FAK-Y397, but not FAK-Y861, reduces ERG and DLL4 expression levels together with concomitant aberrations in vascular patterning. Rapid immunoprecipitation mass spectrometry of endogenous proteins identified that endothelial nuclear-FAK interacts with the deubiquitinase USP9x and the ubiquitin ligase TRIM25. Further *in silico* analysis confirms that ERG interacts with USP9x and TRIM25. Moreover, ERG levels are reduced in FAK^KO^ ECs via a ubiquitin-mediated post-translational modification programme involving USP9x and TRIM25. Re-expression of ERG *in vivo* and *in vitro* rescues the aberrant vessel-sprouting defects observed in the absence of ECFAK. Our findings identify ECFAK as a regulator of retinal vascular patterning by controlling ERG protein degradation via TRIM25/USP9x.

## Abstract (precis)

**Summary:** Coupling of FAK with TRIM25/USP9x controls ERG degradation in endothelial cells, thus determining vascular patterning in the retina.

## INTRODUCTION

The molecular regulation of vascular patterning involves precise control of endothelial cell (EC) proliferation, migration and spatial/temporal organisation within growing blood vessels during angiogenic sprouting. Although vascular endothelial growth factor (VEGF) A is a major regulator of vessel patterning, how VEGF signalling intersects with other molecular networks governing these processes *in vivo* is still under investigation (Bautch, 2012). Among the E-26 transformation-specific (ETS) family members, the transcription factor ETS-related gene (ERG) is a central regulator of EC specification and homeostasis in angiogenesis (Shah et al., 2016). Whereas downstream of VEGF signalling by the Delta-like 4 (DLL4) Notch ligand coordinates the dynamic spatial competition between ECs for a leading position at the tip of a vessel sprout, a process that is thought to be essential in promoting correct patterning (Blanco and Gerhardt, 2013; Jakobsson et al., 2010), the transcriptional regulation of DLL4 expression has been reported to be associated with ERG levels and activity (Wythe et al., 2013; Fish et al., 2017; Shah et al., 2017). However, identification of upstream molecular regulators of ERG expression and function in EC migration and angiogenesis is underexplored.

Focal adhesion kinase (FAK), also known as protein tyrosine kinase 2 (PTK2), is a non-receptor protein tyrosine kinase that acts on downstream cell surface receptors, including VEGF receptors, to transduce their signals, and has been implicated as being essential in EC migration, proliferation, survival and developmental angiogenesis (Mitra et al., 2005; Schaller, 2010; Braren et al., 2006; Shen et al., 2005). ATP binding to the FAK-kinase domain induces phosphorylation at tyrosine (Y) 397 and subsequent phosphorylation at Y861. ECFAK-kinase dead mutant embryos display, and global loss of FAK exon 15, which encodes the Y397 region of FAK, results in, severe vascular defects (Corsi et al., 2009; Zhao et al., 2010; Lim et al., 2010), whereas phosphorylation of Y861 is proposed to be required for *in vitro* VEGF-induced angiogenesis (Abu-Ghazaleh et al., 2001). Despite these results, the specific roles of ECFAK kinase activity and phosphorylation at FAK-Y397 and FAK-Y861 in vascular patterning *in vivo* are not clear. To date, most ECFAK research has focussed on its role at focal adhesions and its cytoplasmic signalling functions. More recently, FAK was described to translocate from the cytoplasmic compartment to the nucleus (Lim, 2013). Although FAK appears to be able to regulate the expression of target genes by binding to transcription factors in several cell types, this has not been extensively studied in ECs and its mechanisms are not well understood. Thus, determining how FAK expression and activity might regulate ERG could reveal the molecular orchestration of vascular patterning.

In this study, we show that nuclear ECFAK acts as an upstream regulator of ERG. ERG expression, via ubiquitin-regulated post-translational modifications, is controlled by nuclear ECFAK association with the deubiquitinase USP9x and the ubiquitin ligase TRIM25. The loss of ECFAK expression and of its kinase activity, or ECFAK phosphorylation at Y397, but not at Y861, all disrupt vessel patterning *in vivo* with a concomitant reduction in ERG and DLL4 expression. EC-tip cell positioning and migration are also abrogated by loss of ECFAK expression, kinase activity or phosphorylation of FAK at Y397, whereas the overexpression of ERG *in vivo* and *in vitro* rescues the loss of FAK-knockout (KO) phenotypes. Overall, our findings identify that nuclear ECFAK regulates TRIM25 and USP9x in the control of ERG expression, providing a FAK-meditated mechanism for vascular patterning control.

## RESULTS

### ERG expression and vascular patterning are controlled by endothelial cell FAK

**Fig. 1.**
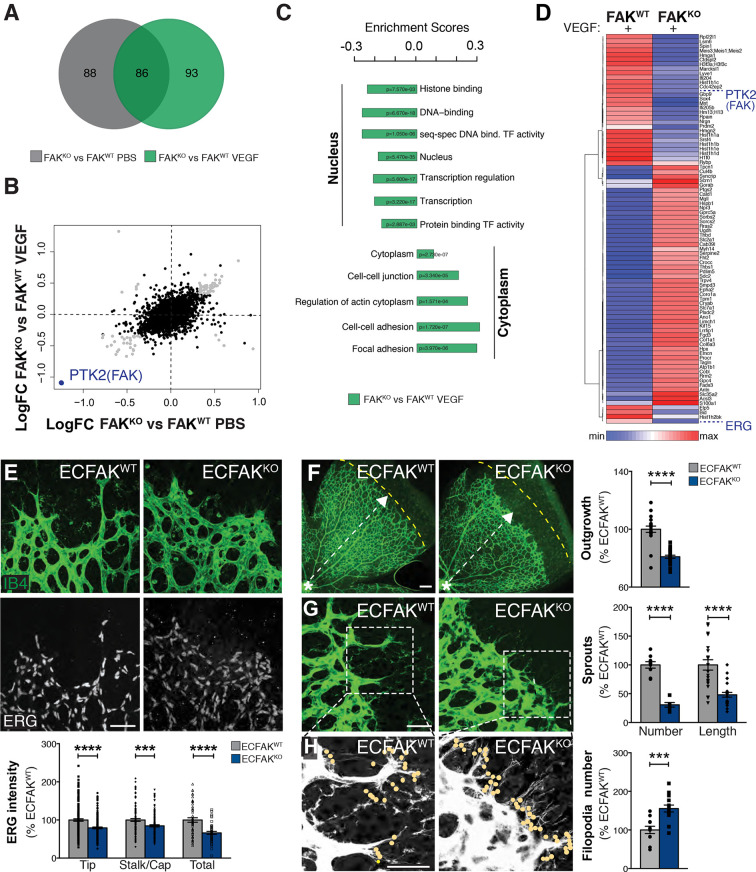
**ERG transcription factor expression is decreased in the absence of ECFAK*.*** (A) Venn diagram depicting DE peptides (*P*<0.05, Benjamini–Hochberg test) from liquid chromatography-tandem mass spectrometry (phospho)proteomic analysis of FAK^KO^ versus FAK^WT^ primary ECs treated with PBS or VEGF. (B) Scatterplot of the logFC of all detected proteins, confirming the knockdown of PTK2 (FAK). (C) Protein category enrichment analysis of FAK^KO^ versus FAK^WT^ ECs after VEGF treatment. Selected GOBP, GOCC, GOMF, KEGG and UniProt keyword terms are displayed with the Benjamini–Hochberg corrected *P*-values. (D) Hierarchical clustering heatmap of 86 common DE proteins. Each column is the mean of the log2 normalised values from each biological replicate (*n*=2) for FAK^WT^ and FAK^KO^ genotypes in the VEGF condition. (E) Representative confocal images of ERG/IB4 double-stained immunofluorescence of nuclear ERG levels in Tip, Stalk/Cap (capillary) and total ECs in P6 ECFAK^WT^ and ECFAK^KO^ retinal angiogenic fronts, and quantification of ERG levels in ECFAK^WT^ and ECFAK^KO^ retina vasculature. (F) Vascular outgrowth in P6 ECFAK^WT^ and ECFAK^KO^ retinal angiogenic fronts. Dashed yellow line indicates the mean angiogenic front distance in P6 ECFAK^WT^. Asterisk indicates the optic stalk, whereas the white dashed arrow indicates vascular outgrowth. (G) Sprout number and length in P6 ECFAK^WT^ and ECFAK^KO^ retinal angiogenic fronts. (H) Number of filopodia in P6 ECFAK^WT^ and ECFAK^KO^ retinal angiogenic fronts (dots indicate filopodial extensions). Data are mean±s.e.m. for *n*=4-6 mice per genotype, analysed using Student's *t*-test (E-H), with ***P<0.001; ****P<0.0001. Scale bars: 50 µm.

FAK flox/flox mice were crossed with mice expressing a tamoxifen-inducible Cre deleter (iCreERT2) driven by an EC-selective platelet-derived growth factor subunit B (*Pdgfb*) promoter (*Pdgfb-iCre*ERT2) (Tavora et al., 2010). Mice without Cre were used as controls (*Pdfgb-iCre*ERT-;FAK^fl/fl^). To induce EC-restricted FAK deletion, we injected 4-hydroxytamoxifen (4-OHT) from postnatal day (P) 1 to P2 and isolated primary ECs from lungs at P10-P14. Molecular signalling events modulated by ECFAK loss, in the context of VEGF stimulation, were examined to identify putative regulators of vascular patterning. Using isobaric tandem mass tagging (TMT)-based quantitative proteomic and phosphoproteomics (Fig. S1A,B), 86 proteins were commonly differentially expressed (DE) between wild-type (FAK^WT^) and FAK^KO^ ECs in control (PBS) and VEGF-stimulated conditions (Fig. 1A). Loss of ECFAK (PTK2) was confirmed and pointed towards putative VEGF-stimulated pathways regulated by ECFAK (Fig. 1B). Specifically, protein category enrichment analysis revealed that FAK deficiency caused an increase in protein categories that reflect cytoplasmic canonical FAK functions, but a decrease in protein categories that reflect nuclear non-canonical FAK functions (Fig. 1C; Fig. S1C,D). Furthermore, hierarchical clustering of the common significantly DE proteins verified the loss of FAK in FAK^KO^ ECs and identified a significant downregulation of ERG in FAK^KO^ ECs after VEGF stimulation (Fig. 1D). Protein category enrichment analysis of the phosphoproteome revealed a similar decrease in nuclear non-canonical FAK functions (Fig. S1E-G). ERG expression levels were reduced in primary FAK^KO^ lung ECs (Fig. S2A-D). Together, these findings established that loss of ECFAK modulates ERG protein expression.

Previous research showed that ERG has a role in retinal angiogenesis (Birdsey et al., 2015; Shah et al., 2017; Fish et al., 2017), a well-established model used to study sprouting and vascular patterning *in vivo* (Blanco and Gerhardt, 2013). Corroborating our previous results, a significant reduction in *in vivo* ERG expression was observed in ECFAK^KO^ retinal vasculature tip cells, stalk cells and total vascular front compared with ECFAK^WT^ controls (Fig. 1E; Fig. S2E). Furthermore, the morphology of retinal vessels in ECFAK^KO^ mice was significantly compromised, showing reduced vascular outgrowth, EC proliferation and number of sprouts and sprout length, together with an increased number of filopodial extensions (Fig. 1F; Fig. S2F). Overall, the loss of ECFAK expression resulted in reduced ERG expression *in vivo* and *in vitro* and this correlated with aberrant retinal vascular patterning.

**Fig. 2.**
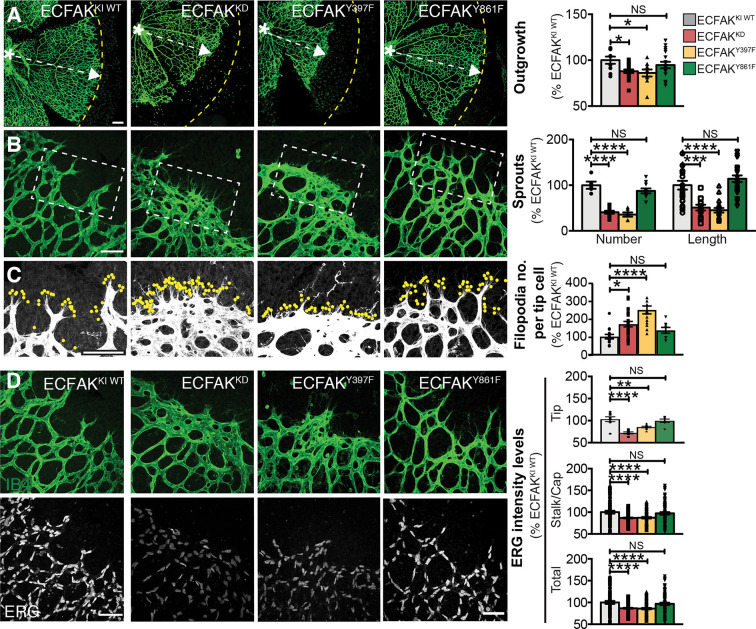
**ERG levels and vascular patterning are reduced by impaired ECFAK-kinase activity and nonphosphorylation at Y397.** (A) Representative confocal images of IB4-stained P6 ECFAK^WTKI^, ECFAK^KD^, ECFAK^Y397F^ and ECFAK^Y861F^ retinal angiogenic fronts with outgrowth quantification. Dashed yellow line indicates mean angiogenic front distance in P6 ECFAK^WTKI^. Asterisks indicate the optic stalk, whereas dashed white arrows indicate vascular outgrowth. (B) Examination of vascular sprout numbers and length and (C) filopodia numbers (dots indicate filopodia extensions) in IB4-stained P6 ECFAK^WTKI^, ECFAK^KD^, ECFAK^Y397F^ and ECFAK^Y861F^ retinal angiogenic fronts. (D) ERG/IB4 double-stained immunofluorescence images in ECFAK^WTKI^, ECFAK^KD^, ECFAK^Y397F^ and ECFAK^Y861F^ retinal vasculature with quantification of nuclear ERG protein levels in tip, stalk/capillary (Cap) and total ECs in P6 ECFAK mutant retinal angiogenic fronts. Data are mean±s.e.m. for *n*=3-6 mice per genotype. One-way ANOVA was used in all panels, with *P<0.05; **P<0.01; ***P<0.001; ****P<0.0001; NS, not significant. Scale bars: 50 µm.

To determine whether FAK kinase activity and/or phosphorylation of FAK at Y397 and/or Y861 regulates ERG expression and retinal angiogenesis, we next examined the effect of inducible EC-specific point mutations in: (1) FAK-kinase dead mutant (ECFAK^KD^) mice, in which FAK catalytic activity is abolished by a K454R mutation; (2) nonphosphorylatable Y397 (ECFAK^Y397F^) mice; and (3) nonphosphorylatable Y861 (ECFAK^Y861F^) mice (Alexopoulou et al., 2017; Pedrosa et al., 2019; Newport et al., 2022). Wild-type (WT) FAK-knock-in mice (ECFAK^KIWT^) were used as controls. The Cre-inducible system for mutant FAK expression was generated by designing mutant chicken-FAK constructs (preceded by a STOP sequence flanked by loxP sites) that were targeted to the ubiquitous Rosa26 locus (R26FAKKI/KI) (Tavora et al., 2014). R26FAKKI/KI mice were bred with the inducible endothelial-specific FAK^KO^ mice we used previously. Administration of 4-OHT led to simultaneous deletion of the endogenous mouse *Fak* (*Ptk2*) and the neoSTOP cassette, thus allowing the homozygous expression of the mutated chicken-FAK transgene; WT (KIWT), KD, nonphosphorylatable Y397 (Y397F) or nonphosphorylatable Y861 (Y861F) knock-in-chicken-FAK expression specifically in ECs generated ECFAK^KIWT^, ECFAK^KD^, ECFAK^Y397F^ and ECFAK^Y861F^ mice, respectively. Analysis of the retinal vasculature at P6 revealed that vascular outgrowth, numbers and length of sprouts were all reduced in ECFAK^KD^ and ECFAK^Y397F^ but not in ECFAK^Y861F^ mutants (Fig. 2A,B). Additionally, the number of filopodia at the vascular front increased significantly in both ECFAK^KD^ and ECFAK^Y397F^, but not in ECFAK^Y861F^ mice, compared with ECFAK^KIWT^ controls (Fig. 2C). Concomitantly, ERG expression was reduced in ECFAK^KD^, ECFAK^Y397F^, but not ECFAK^Y861F^ retinal vasculature (Fig. 2D). These *in vivo* data were also supported by reduced *Erg* mRNA levels in FAK^KD^ and FAK^Y397F^, but not in FAK^Y861F^, primary ECs from P6 pups (Fig. S2G). EC-specific mutant FAK-knock-in system validation was performed (Fig. S3). Together, these data suggest that physiological FAK expression, in particular, FAK-kinase activity and phosphorylation at Y397, are required for ERG regulation in the control of retinal vascular patterning.

### DLL4/Notch signalling and sprouting depend on ECFAK

**Fig. 3.**
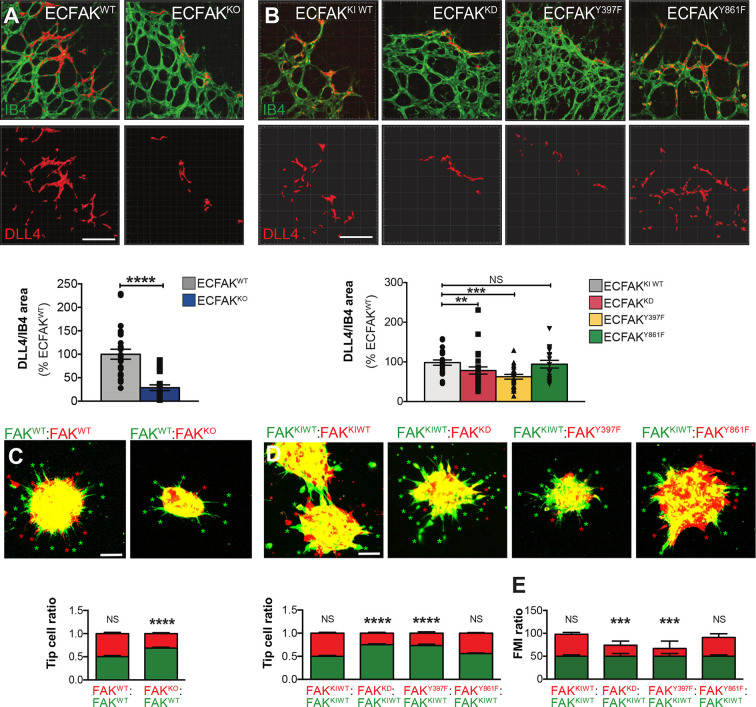
**DLL4 expression and tip cell positioning are associated with ECFAK deficiency, FAK-kinase activity and phosphorylation at FAK-Y397*.*** Imaris 3D visualisation of DLL4/IB4 double-stained P6 retinal angiogenic fronts and DLL4 quantification *in vivo* in (A) ECFAK^WT^ and ECFAK^KO^ and (B) ECFAK^KIWT^, ECFAK^KD^, ECFAK^Y397F^ and ECFAK^Y861F^ mice. *n*=4-6 mice in A and 3-6 mice in B. Data were analysed with a Student's *t*-test in A and one-way ANOVA in B. (C) Confocal images and quantification of *in vitro* sprouting competition assays of differentially labelled (green or red dye) FAK^WT^ and FAK^KO^ or (D) FAK^KIWT^, FAK^KD^, FAK^Y397F^ and FAK^Y861F^ mutant EC lines. Asterisks indicate ECs at the tip position. *n*=10-20 spheroids per condition from two independent experiments. (E) VEGF-induced migration competition assays *in vitro* between FAK^KIWT^ (green) and FAK^KIWT^, FAK^KD^, FAK^397F^ or FAK^861F^ (red) primary mouse lung ECs. Forward Migration Index (FMI) quantifications are shown. *n*=3 independent experiments analysing 31-60 cells per condition in each. Data are mean± s.e.m. analysed using a two-way ANOVA; *P<0.05; **P<0.01; ***P<0.001; ****P<0.0001; NS, not significant in A-E. Scale bars: 50 µm.

Given that transcriptional regulation of DLL4 has been associated with ERG activity (Wythe et al., 2013; Fish et al., 2017; Shah et al., 2017), we next sought to investigate the expression of DLL4 in ECFAK-mutant mice. Whereas DLL4 decorated the ECs at the vascular front in ECFAK^WT^ retinas, a significant reduction in DLL4 protein expression levels was observed in ECFAK^KO^, ECFAK^KD^ and ECFAK^Y397F^, but not ECFAK^Y861F^, retinas (Fig. 3A,B; Fig. S4A-F). In line with this, transcripts for *Fak* and several Notch signalling effectors, including *Dll4*, *Hey1*, *Hey2* and *Nrarp*, were reduced in FAK^KO^ ECs (Fig. S4G). Furthermore, Notch receptor intracellular domain (NICD) expression levels, which reflect ligand-dependent activation of Notch signalling (Blanco and Gerhardt, 2013), were also reduced in FAK^KO^ ECs (Fig. S4G-I). Together, these data indicate that the loss of ECFAK expression, ECFAK-kinase activity and phosphorylation at FAK-Y397 reduce DLL4 expression similarly to the reduction seen in ERG expression.

To investigate whether the ECFAK-mediated control of ERG and DLL4 expression was associated with functional changes in EC spatial organisation within vascular sprouts, fluorescent colour-dye prelabelled control primary ECs in green (FAK^WT^ or FAK^KIWT^) and prelabelled primary FAK-mutant ECs in red (FAK^WT^, FAK^KIWT^, FAK^KO^, FAK^KD^, FAK^Y397F^ or FAK^Y861F^) were used in 2D and 3D competition assays. Results indicated that FAK^KO^, FAK^KD^ and FAK^Y397F^ ECs, but not FAK^Y861F^ ECs, were unable to locate at the tip cell position in the 3D sprouting competition assays (Fig. 3C,D). Additionally, both FAK^KD^ and FAK^Y397F^ ECs, but not FAK^Y861F^ ECs, showed a significant decrease in 2D directed chemotaxis behaviour in the presence of FAK^KIWT^ cells (Fig. 3E). Overall, our data suggest that the reduction in ERG and DLL4, together with the reduction in FAK levels and, importantly, FAK-kinase activity as well as phosphorylation at Y397, but not at Y861, are involved in establishing endothelial tip-stalk cell fate and/or its precise spatial organisation of ECs within vessel sprouts.

### Nuclear ECFAK interactome with ERG-associated USP9x and TRIM25

We next sought to identify the molecular mechanism of ERG regulation by FAK in ECs. FAK has been reported to function in both the cytoplasm and the nucleus, in which it has DNA-binding roles (Lim, 2013). Relevant to our study, published work demonstrates that ERG-dependent dynamic induction of DLL4 transcription in human umbilical vein ECs (HUVECs) *in vitro* peaks at 30 min of VEGF stimulation (Fish et al., 2017). Therefore, we analysed nuclear and cytoplasmic primary mouse EC fractions after VEGF stimulation for 30 min and found that full-length FAK^WT^ translocated to the nucleus, as did phosphorylated FAK in FAK^KIWT^, FAK^Y861^ FAK^KD^ and FAK^Y397F^ ECs (Fig. S5A-C), confirming previous reports of the nuclear translocation of FAK despite the lack of its kinase function (Lim, 2013).

**Fig. 4.**
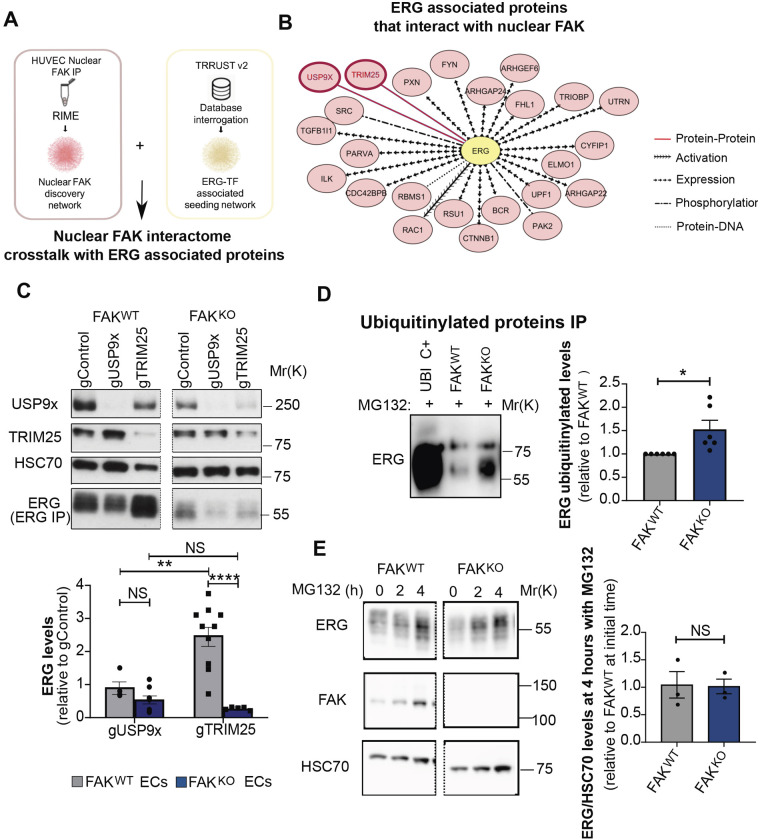
**Nuclear ECFAK interacts with USP9x and TRIM25 to regulate ERG levels.** (A) Interactome network overlapping of nuclear ECFAK interactome (RIME) with ERG-associated transcription factors (TRRUST v2). *n*=25 biological replicates. (B) Molecules associated with ERG were used to seed a functional interaction network (QIAGEN's IPA) with nuclear FAK interactors under PBS control and nuclear FAK interactions under VEGF-stimulated conditions. The legend indicates the interaction type. The direction of the arrowheads denotes the direction of association. The settings used for network generation were those for which interactions were experimentally observed upstream or downstream of the transcription factor and were direct. The Benjamini–Hochberg test was used to select interactors with *P*<0.05. (C) Western blot of USP9x and TRIM25 in gControl, gUSP9X and gTRIM25 CRISPR/Cas9-depleted FAK^WT^ and FAK^KO^ ECs. Lower panel shows ERG immunoprecipitation followed by ERG western blot. Images of the immunoblots shown were cropped and correspond to the same membrane and exposure. Bar charts indicate ERG levels relative to gControl for each genotype. *n*=3-10 replicates from 3-5 independent experiments, analysed with a two-way ANOVA. (D) Immunoprecipitation for ubiquitylated proteins followed by western blotting for ERG in FAK^WT^ and FAK^KO^ ECs treated with the MG132 proteasome inhibitor for 4 h. *n*=6 independent experiments. Data analysed using the Wilcoxon Mann–Whitney rank test. (E) Western blot analysis for ERG and FAK in FAK^WT^ and FAK^KO^ ECs treated with MG132 for 0, 2 and 4 h. Images of the immunoblots shown were cropped and correspond to the same membrane and exposure. Quantification of ERG/HSC70 level ratios at 4 h after MG132 treatment compared with FAK^WT^ ECs at time zero. *n*=3 independent experiments. Data are mean± s.e.m., analysed using a Student's *t*-test, with *P<0.05; **P<0.01; ****P<0.0001, NS, not significant in all instances.

Rapid immunoprecipitation mass spectrometry of endogenous proteins (RIME) (Mohammed et al., 2016) was applied to HUVECs treated with either PBS or VEGF to analyse FAK interactors (Fig. S6A). From the approximately 500 nuclear FAK-specific interactors identified using TRRUSTv2 and QIAGEN database interrogation, 24 of the nuclear FAK-specific interactors identified by RIME analysis were experimentally predicted to interact with ERG (Fig. 4A,B). These included two protein–protein interactions with: (1) the deubiquitinase USP9x, and (2) the ubiquitin ligase TRIM25, which have both been reported to promote ERG stabilisation and ERG degradation, respectively, in cancer cells (Wang et al., 2014; Wang et al., 2016).

### ECFAK regulates ERG expression via ubiquitylation

To test the functional requirement for USP9x and TRIM25 in ERG expression in FAK^WT^ and FAK^KO^, USP9x and TRIM25 were depleted in FAK^WT^ and FAK^KO^ ECs using a CRISPR/Cas9 gene-editing approach. USP9x depletion (gUSP9x) reduced total ERG expression levels compared with genotype-matched gControls, in both FAK^WT^ and FAK^KO^ ECs (Fig. 4C). In contrast, TRIM25 depletion (gTRIM25) caused an upregulation of total ERG expression level compared with gControl in FAK^WT^ cells, but a downregulation of ERG expression level compared with ERG expression level in gControl FAK^KO^ ECs. Given that USP9x is a deubiquitinase and TRIM25 a ubiquitin ligase, the overall ubiquitylation status of ERG was next assessed to test a possible link with ERG expression regulation by proteosomal degradation. Immunoprecipitation of ubiquitylated proteins (UBI) in the presence of the proteasome inhibitor MG132, which is known to repress protein degradation allowing accumulation of ubiquitylated proteins, followed by western blotting for ERG, showed that ubiquitylation of ERG was enhanced significantly in FAK^KO^ compared with FAK ^WT^ ECs, suggesting that FAK modulates ERG protein degradation (Fig. 4D). Additionally, treatment with MG132 for 4 h also rescued the levels of ERG in FAK^KO^ ECs to similar levels as in FAK^WT^ ECs (Fig. 4E). Thus, FAK contributes to regulating protein expression levels of ERG through modulation of ubiquitylation and deubiquitylation by USP9X and TRIM25, respectively.

### ERG re-expression restores DLL4 levels and FAK-dependent vessel patterning phenotypes

**Fig. 5.**
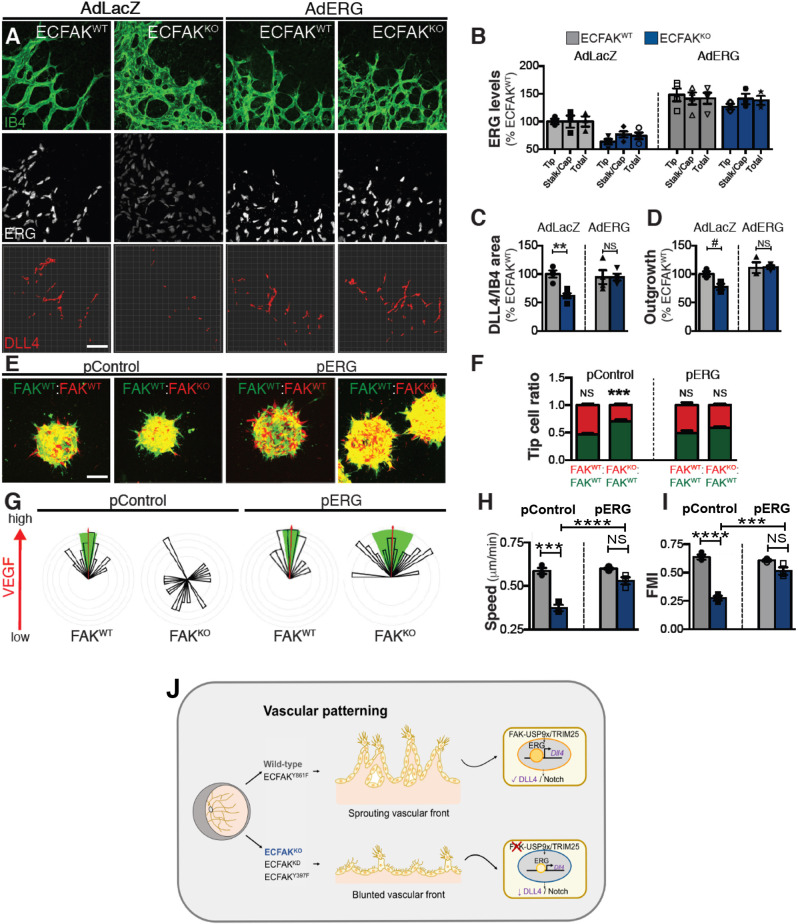
**ERG re-expression in ECFAK^KO^ restores DLL4 levels and vascular outgrowth *in vivo* and tip cell positioning and directed chemotactic migration *in vitro*.** (A) IB4/ERG/DLL4 triple-stained retinal fronts and quantification in Ad-*lacZ* or Ad-ERG P6 ECFAK^WT^ and ECFAK^KO^ pups. (B) ERG and (C) DLL4 in Ad-*lacZ* or Ad-ERG P6 ECFAK^WT^ and ECFAK^KO^ pups transduced *in vivo*. (D) Vascular outgrowth analysis. *n*=3 mice per genotype. (E) Representative images and (F) quantification of a sprouting competition assay of FAK^WT^ and FAK^KO^ ECs nucleofected with control (pControl) or ERG (pERG) plasmids *in vitro. n*=10-20 spheroids per condition. (G) Dunn chamber chemotaxis, pControl- or pERG- FAK^WT^ and FAK^KO^ ECs. (H) Speed and (I) FMI analysis*. n*=3 independent experiments analysing 22-44 cells per condition. (J) Schematic of how ERG regulation by ECFAK affects blood vessel development and patterning. Data are mean±s.e.m., analysed using a two-way ANOVA, with **P<0.01; ***P<0.001; ****P<0.0001; # 0.0642; NS, not significant. Scale bars: 50 µm.

The functional requirement for ERG in FAK-regulated vascular patterning was assessed next. ECFAK^WT^ and ECFAK^KO^ newborn pups were injected at P1 (Klose et al., 2015) with an ERG adenovirus, either Ad-ERG-V5 (Sperone et al., 2011) or Ad-*lacZ*-V5 as a control. Although Ad-*lacZ*-V5 infection had no overt effects, restoration of ERG expression by Ad-ERG-V5 infection resulted in a significant rescue of both DLL4 expression and vessel outgrowth in ECFAK^KO^ mice. Likewise, Ad-ERG-V5 infection of ECFAK^KD^ and ECFAK^Y397F^ mutants restored both ERG and DLL4 levels back to those found in control pups, and also restored the vascular outgrowth phenotypes in these mice (Fig. S7A-D). Conversely, depleting ERG expression, using ERG-targeted small interfering (si)RNA *in vitro*, in primary ECs from ECFAK^Y861F^ newborns, showed a reduction in *Dll4* expression but not in that of *Pecam1*, confirming a regulation of DLL4 by ERG in FAK^Y861F^ mutant ECs (Fig. S7E). Validation of the experimental system was confirmed using Ad-Cre viruses in FAK^flox/flox^ pups (Fig. S8). Lastly, ERG overexpression (pERG) (Sperone et al., 2011) in FAK^KO^ ECs (pERG-FAK^KO^) rescued the compromised tip cell position phenotype caused upon FAK deletion (pControl-FAK^KO^) (Fig. 5E,F) and the reduced VEGF-mediated migratory response phenotype in these cells (Fig. 5G-I). Altogether, ERG re-expression restores DLL4 levels, EC migration, tip cell positioning and FAK-mediated vessel patterning phenotypes *in vivo* and *in vitro*.

## DISCUSSION

Here, we identified a fundamental mechanism in the control of vascular patterning via EC nuclear FAK-USP9x and -TRIM25 interactions in the regulation of ERG expression by ubiquitylation. ERG expression is controlled by ECFAK and its kinase activity. Our data indicate that nuclear ECFAK-USP9x/TRIM25-ERG-DLL4 regulates the control of Notch activity, thus governing endothelial tip and stalk cell positioning and vascular patterning (Blanco and Gerhardt, 2013; Jakobsson et al., 2010; Ubezio et al., 2016; Shah et al., 2017) (Fig. 5J).

The current understanding of the regulation of ERG expression is still in its infancy. *ERG* is also known to be fused to transmembrane protease serine 2 (TMPRSS2) in prostate cancer cells, resulting in ERG overexpression. Although recent studies revealed that E3 ubiquitin ligases, such as SPOP and TRIM25, interact specifically with, and promote the ubiquitination and degradation of, oncogenic ERG (Wang et al., 2016; Gan et al., 2015); whether either SPOP or TRIM25 regulates physiological ERG levels in ECs was unclear. A recent study showed that casein kinase I phosphorylates ERG to promote SPOP/ERG interaction and, thus, degradation of oncogenic ERG (Gan et al., 2015). Interestingly, it has been shown in prostate cancer cells that the deubiquitinase USP9X interacts with ERG and promotes its stabilisation to decrease prostate cancer cell proliferation (Wang et al., 2014). Our data identify that nuclear ECFAK interacts with TRIM25 and USP9X, which is relevant in the regulation of ERG proteasomal ubiquitylation and the control of ERG protein levels in ECs and vessel patterning.

Our study also suggests that loss of ECFAK or its kinase activity, or phosphorylation at FAK-Y397 are each sufficient to reduce sprouting angiogenesis in the mouse retina while increasing filopodial extensions. The basis of these vascular-patterning defects are associated with not only reduced ERG expression, but also reduced DLL4 expression and reduced EC migration and competition for the tip cell position at the distal end of vessel sprouts. Thus, the ECFAK phenotype appears to recapitulate some, but not all, of the phenotypes in DLL4-haploinsufficient mice or those treated with pharmacological inhibitors of the DLL4/Notch pathway. For example, the increase in filopodia observed in our mutant mice was also observed in DLL4-haploinsufficient mice (Blanco and Gerhardt, 2013; Jakobsson et al., 2010). However, the blunted vascular front with associated reduction in vessel sprouts was not expected. These apparent differences might be explained by a recent report showing that DLL4 expression is in fact dynamic within ECs of the vascular sprout, and that synchronisation of DLL4 oscillations switches from a sprouting to a nonsprouting expanding phenotype (Ubezio et al., 2016). Most interestingly, the activity of transcription factors can be influenced by their own turnover, as shown for estrogen receptor-alpha or c-MYC (Gierisch et al., 2016). Given that ECFAK affects ERG expression and regulation of DLL4 (Wythe et al., 2013; Fish et al., 2017; Shah et al., 2017), this likely represents a mechanism for the regulation of vascular patterning in the absence of ECFAK or its kinase functions.

Our study not only advances our understanding of the basic mechanisms and concepts of vascular biology, but may also have clinical relevance to FAK expression in some human eye disorders. Pathological angiogenesis is the underlying basis of many ocular diseases, such as retinopathy of prematurity, proliferative diabetic retinopathy and wet age-related macular degeneration. Published work showed that *FAK* expression levels change during ageing of human neural retina (Cai et al., 2012) and FAK and phosphorylation levels are overexpressed in human uveal melanomas (Faingold et al., 2014). However, the specific role of endothelial FAK and/or phosphorylation level are unknown. Norrie disease and familial exudative vitreoretinopathy (FEVR) are both hereditary eye disorders characterised by aberrant and incomplete retinal vascular development, and are associated with mutations in *Fzd4* (Junge et al., 2009), which has been linked to ERG expression in other angiogenesis-associated disease (Gupta et al., 2010). Moreover, ERG expression is lost in other pathological diseases, such as atherosclerosis (Sperone et al., 2011), chronic liver disease (Dufton et al., 2017) and pulmonary arterial hypertension (Looney et al., 2017). Whether a role of endothelial FAK in ERG expression regulation is implicated in those contexts remains to be established.

## MATERIALS AND METHODS

### Study and experimental design

This was a controlled laboratory study using mice and involved *in vivo* and *in vitro* techniques. Mice were administered 4-OHT to induce chicken FAK (wild type and mutant) expression in ECs. Whole mouse litters from each strain/colony were treated and processed. Mice with specific genotypes were enrolled into the experimental study. The treatment of pups with 4-OHT or adenoviruses (to induce deletion/mutation or expression of target proteins, respectively), as well as the weighing and dissection of tissues were all blinded procedures. Imaging and analyses were all performed in a blinded fashion. Samples sizes were based on previous experience and published work. Samples from pups and/or cells in which transgenic inductions (deletions/knock-in) failed (e.g. endogenous FAK deletion/mutated FAK ratio was not satisfactory based on mRNA and protein analysis) were not included for analysis. The number (*n*) for experiments, sample size and experimental replicates are stated in the figure legends and Materials and Methods.

### Mice breeding and procedures

FAK conditional-KO and -knock-in mouse models were generated as previously described (Newport et al., 2022; Alexopoulou et al., 2017; Pedrosa et al., 2019; Tavora et al., 2014; Tavora et al., 2010). FAK^fl/fl^ mice were crossed with an inducible endothelial-specific Cre transgenic deleter line, *Pdgfb-iCre*ERT (Claxton et al., 2008), to generate *PdgfbiCre*ERT;FAK^fl/fl^ and additionally crossed with R26FAK^KI/KI^: R26FAK^KIWT/KIWT^, R26FAK^KD/KD^, R26FAK^Y397F/Y397F^ and R26FAK^Y861F/Y861F^ to generate *PdgfbiCre*ERT;FAK^fl/fl^; R26FAK^KI/KI^ mice (see Table 1 for mouse nomenclature). All mice used were maintained on a mixed C57BL6/129 genetic background. The endothelial FAK-deletion and FAK-KI mutations were induced by treatment with 4-OH-tamoxifen (4-OHT, H7904, Sigma-Aldrich; in a mixed solution of ethanol/sunflower oil of 1:10). Unless otherwise stated, 20 µl/g of a solution at 3.3 mg/ml was injected intragastrically in newborn mice at P1 and P2. Mice were humanely sacrificed and examined at P6 and P10-14. All experiments were conducted using littermate controls. Husbandry and experiments were performed in accordance with the UK Animals (Scientific Procedures) Act 1986 regulations, under Home Office Project License No. PF220CE02.

**Table 1.**
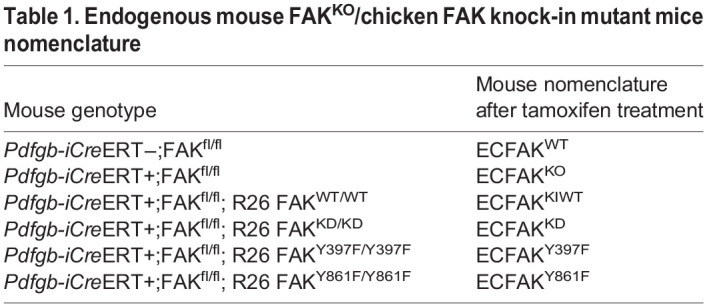
Endogenous mouse FAK^KO^/chicken FAK knock-in mutant mice nomenclature

### Genotyping PCR

Genotyping of *PdgfbiCre*ERT;FAK^fl/fl^ and *PdgfbiCre*ERT;FAK^fl/fl^; R26FAK^KI/KI^ mice was performed with DNA isolated from ear or tail snips by using primers and protocols previously published (Tavora et al., 2014).

### Adenovirus and rescue experiments *in vivo*

ERG expression was carried out using a V5-tagged ERG-3 adenovirus (Ad-ERG), described previously (Sperone et al., 2011). A β-galactosidase adenovirus (Ad-*lacZ*) was used as control. Large-scale production of high-capacity adenoviruses was performed as previously published (Wang et al., 2003). Newborn mice from *PdgfbiCre*ERT;FAK^fl/fl^; *Pdgfb-iCre*ERT;FAK^fl/fl^; R26FAK^KD/KD^ and *PdgfbiCre*ERT;FAK^fl/fl^; R26FAK^Y397F/Y397F^ were intraperitoneally injected with 1×10^8^ PFU at P1. Subsequently, the pups were intragastrically injected with 4-OHT (solution/volumes as described above) at P2 and P3 to induce endogenous FAK deletion and, for the knock-in strains, simultaneous chicken FAK-mutated expression. At P6, pups were sacrificed by cervical dislocation and tissues, including whole eyes, lungs and livers, were collected for analysis. Two litters for each adeno-treated group per mice strain were used to obtain no fewer than four mice per condition.

### Whole-mount immunofluorescence analysis of mouse retinas

Whole eyes were collected from ECFAK^KO^ and ECFAK^KI^ mice at P6. Retinas were dissected and processed for whole-mount immunofluorescence staining as described previously (Pitulescu et al., 2010). Briefly, whole eyes were fixed with freshly prepared 4% paraformaldehyde (PFA) in PBS for up to 6 h at 4°C on a rocking platform. Retinas were dissected and blocked/permeabilised in Claudio Blocking Buffer (CBB) (Franco et al., 2015) for 2 h at room temperature, then incubated overnight on a rocking platform at 4°C in 1:1 CBB plus rabbit-anti-ERG (1:200, ab9251, Abcam) and goat anti-DLL4 antibodies (1:100, AF1389, R&D). After washing several times in PBS at room temperature, retinas were incubated overnight at 4°C in 1:1 CBB plus donkey anti-rabbit AlexaFluor 647 and donkey-anti-goat AlexaFluor 555 antibodies (1:300, A-31573 and A-21432, respectively, Thermo Fisher Scientific). After washing several times in PBS at room temperature, retinas were post-fixed in 1% PFA for 2 min, rinsed in PBS, washed twice in PBLEC buffer (1% Triton X-100, 0.1 mM MgCl_2_, 0.1 mM CaCl_2_, 0.1 mM MnCl_2_ in PBS, pH 6.8), reblocked in 1% BSA and 0.5% Triton X-100 in PBS for 1 h and incubated overnight in PBLEC plus biotinylated *Griffonia simplicifolia* Lectin I Isolectin B4 (IB4, 1:25, B-1205, Vector Laboratories). After further washes in PBS, retinas were incubated overnight on a rocking platform in 0.5% BSA and 0.3% Triton X-100 in PBS plus streptavidin AlexaFluor 488 (1:100, S11223, Thermo Fisher Scientific). Retinas were washed several times in PBS, further stained with Hoechst 33342 in PBS (1:5000, H3570, Thermo Fisher Scientific) for 30 min at room temperature, rinsed in PBS twice, post-fixed in 1% PFA for 2 min, further washed in PBS and flat-mounted on glass microscope slides using ProLong Gold anti-fade mounting medium (P36930, Thermo Fisher Scientific). Confocal microscopy imaging was carried out using ZEISS LSM710 and LSM 880 AiryScan microscopes.

### EdU labelling of proliferating ECs

For detection of proliferating ECs *in vivo* in ECFAK^KO^ mice, a stock of 1 mg of 5-ethynyl-2′-deoxyuridine (EdU) (A10044, Thermo Fisher Scientific) was dissolved in a 10% DMSO/PBS solution; 10 μl of this stock solution/gram of body weight was injected intraperitoneally 4 h before the mice were sacrificed. Whole eyes were collected and processed as described above. Retinas were dissected and permeabilised for 2 h at room temperature with 0.5% Triton X-100 in PBS. EdU-positive cells were detected with the Click-iT EdU AlexaFluor 647 Imaging Kit (C10340, Thermo Fisher Scientific). ECs were detected by ERG and IB4 immunofluorescence staining as described above but using donkey anti-rabbit AlexaFluor 594 as a secondary antibody for ERG detection instead (1:1000, 37121, Thermo Fisher Scientific). Confocal microscopy imaging was carried out using a LSM710 microscope.

### Anti-adenovirus Hexon protein immunohistochemistry

PFA paraffin-embedded liver histological sections from P6 ECFAK^WT^ and ECFAK^KO^ mice that were transduced with Ad-ERG or Ad-*lacZ* were used for Hexon immunostaining. Tissue sections were dewaxed, rehydrated through graded alcohols and pretreated with trypsin enzymatic antigen retrieval solution (ab970, abcam) before immunohistochemistry. Tissue sections were stained using adenovirus Hexon protein antibodies (1:1000, ab8249, Abcam) and an appropriate secondary HRP-conjugated antibody VECTASTAIN Elite ABC reagent (1:100, PK-6100, Vector Laboratories) with liquid DAB+ substrate. Imaging was performed using an Axiophoto microscope equipped with ZEISS AxioCam HRC and associated AxioVision Rel 4.8 software, using 20×/0.50 Ph2 objectives.

### EC preparations

Primary mouse lung EC (MLEC) isolation from ECFAK^WT^, ECFAK^KO^, ECFAK^KIWT^, ECFAK^KD^, ECFAK^Y397F^ and ECFAK^Y861F^ pups at P6 was performed as previously described (Reynolds and Hodivala-Dilke, 2006) with some modifications. Briefly, lungs from pups that were tamoxifen induced at P1 and P2 to achieve FAK deletion and FAK point-mutation expression were excised at P6. Lungs from pups with the same genotype were pooled (2-4 lungs/genotype), minced and digested for 1 h at 37°C with Collagenase type-1 (17100017, Thermo Fisher Scientific), passed through a 70 µm pore size cell strainer (352350, BD Falcon) into MLEC growth media and plated onto tissue culture plates coated with 0.1% gelatin, 10 µg/ml human plasma fibronectin (FC010, Millipore) and 30 µg/ml collagen (C3867, Sigma-Aldrich) and incubated for overnight at 37°C and 5% CO_2_. A single positive-sort for EC enrichment was performed using rat anti-mouse monoclonal CD102/ICAM2 (1:1000, 3C4 mlC2/4, 555326, BD Pharmingen) and CD31/PECAM1 (1:1000, MEC13.3, 550274, BD Pharmingen) antibodies together and sheep anti-rat Dynabeads (11035, Thermo Fisher Scientific). Unless otherwise stated, MLECs were used freshly isolated without further culture for experiments. MLEC growth media comprised one part Dulbecco's Modified Eagle's Medium low glucose (D-MEM, 21885-025, Gibco), one part Ham's F12 medium (N6658, Sigma-Aldrich), supplemented with 0.1 mg/ml heparin (H3149, Sigma-Aldrich), 100 μg/ml penicillin/streptomycin (P4333, Gibco), 6 mM L-glutamine (25030-081, Gibco), 20% fetal bovine serum (heat inactivated) (AXB 32773, HyClone) and 50 μg/ml endothelial mitogen (J645516, Alfa Aesar). Immortalised MLECs from ECFAK^WT^, ECFAK^KO^, ECFAK^KIWT^, ECFAK^KD^, ECFAK^Y397F^ and ECFAK^Y861F^ mice were described previously (Alexopoulou et al., 2017; Pedrosa et al., 2019; Tavora et al., 2010).

### Immunofluorescence in primary ECs

Freshly isolated primary MLECs from ECFAK^WT^ and ECFAK^KO^ were seeded in MLEC growth media at a density of 20,000 cells/10 mm^2^ onto glass coverslips coated as described above. Cells were left to attach for 4 h-overnight at 37°C and 5% CO_2_, then fixed in 4% PFA for 7 min at room temperature and washed three times in PBS and then permeabilised with 0.1% Triton-X100 in PBS for 10 min; next, the cells were washed three times in PBS and blocked with 1% BSA in PBS for 30 min at room temperature. Primary antibodies were diluted in PBS and incubated for 1 h at room temperature: mouse-anti FAK (Clone 77, 1:100, 610287, BD Pharmingen), rat anti-CD144/VE-cadherin/CDH5 (Clone 11D4.1, 1:100, 555289, BD Pharmingen), goat-anti-DLL4 antibodies (1:100, AF1389, R&D) and rabbit-anti-ERG (1:100, ab9251, Abcam). Cells were washed three times in PBS and incubated with the corresponding secondary antibodies: donkey anti-mouse Alexa Fluor 647, donkey anti-rat Alexa Fluor 488, donkey-anti-goat Alexa Fluor 555 antibodies and donkey anti-rabbit Alexa Fluor 647 (1:250, A-31571, A-31573, A-21208 and A-21432, respectively; Thermo Fisher Scientific) for 30 min at room temperature. After three washes in PBS, the coverslips were mounted onto microscope glass slides using ProLong Gold Antifade reagent with DAPI (P36931, Molecular Probes). Confocal microscopy imaging was carried out using a LSM710 microscope.

### Microscopy and retinal vasculature quantification analysis

Fluorescently labelled samples (flat whole-mount retinas and MLECs) were analyzed using confocal microscopes (LSM710 or LSM880 Airyscan) with objectives 10× with NA 0.45, and oil objectives 40× with NA 1.3 and 63× with NA 1.4. Images were acquired using the multichannel fluorescent in-frame mode, multi-tile and/or *z*-stack modules. Composites from up to four fluorescent channels, maximum intensity projections and tile stitching of images were created and analysed by using Imaris, together with ImageJ and CellProfiler, as indicated below. All quantifications were performed across littermates.

The quantification of various vascular morphometric parameters, such as plexus outgrowth, sprout number and length and filopodial number, was performed manually as previously described (Lobov et al., 2007; Franco et al., 2015; Pitulescu et al., 2010). For filopodia, the number was normalised to the length of the vascular front. The vascular surface area in retinas was quantified as the IB4-positive area from confocal micrographs acquired from all intact quarters of the processed retinas and at a similar distance from the optic stalk, using Fiji software.

Endothelial DLL4 expression was quantified by thresholding the area of the DLL4 signals that were IB4 positive from 40× confocal micrographs acquired from the angiogenic fronts of the retinas. These were expressed relative to the total vascular surface area quantified as described above by IB4. Similarly, DLL4 staining was quantified using Imaris (using area volume).

ERG expression in the tip and stalk/capillary cells was quantified using a combination of a script and pipeline written in-house for Fiji and CellProfiler respectively. The mean ERG expression intensity in each nucleus was calculated by dividing its 3D integrated intensity (after background subtraction) by its equivalent ellipsoid volume. To do so, an intensity integration along the axial (*z*) direction was first obtained using Fiji, by performing a sum projection of the ERG image stacks. The resulting 2D SUM images were then processed using a CellProfiler pipeline to segment and quantify automatically and individually each ERG-positive nucleus, both in terms of intensity and shape. This provided a subsequent lateral intensity integration (*xy*) for each nucleus, as well as the long and short axis length of a fitted ellipse, equivalent in area to the segmented nuclei. The estimated nuclei volumes for each ERG-positive nucleus were then obtained from the volumes of their matching ellipsoids using the standard volume formula 4/3.π.a.b^2^, where a and b are the long and short axis lengths, respectively. For automated differential analysis, the CellProfiler pipeline also enabled the manual selection of ERG-positive nuclei residing in the tip of IB4-positive vasculature, relative to ERG nuclei residing in the vascular stems.

Retinal EC proliferation was examined by quantifying the number of double EdU- and ERG-positive cells (well-known proliferative and EC nuclear markers, respectively) relative to the total ERG-positive EC nuclei present in the angiogenic vascular front from retinas identified by IB4 positivity.

### Quantitative real-time PCR analysis

Freshly isolated primary MLECs and tissues, including retinas, lungs and livers, from ECFAK^WT^, ECFAK^KO^, ECFAK^KIWT^, ECFAK^KD^, ECFAK^Y397F^ and ECFAK^Y861F^ pups at P6 were lysed in RLT buffer (79216, QIAGEN). Total mRNA was isolated using the RNAEasy Mini kit (74104, QIAGEN). Quality control and concentration of samples was carried out using a Nanodrop ND-10000 spectrophotometer. RNA was reverse transcribed using a High Capacity cDNA Reverse Transcription kit (4368814, Applied Biosystems) according to the manufacturer’s instructions. Real-time PCR was performed in a StepOne Plus thermocycler (4376357, Applied Biosystems) using TaqMan Master mix (4369514, Applied Biosystems) and primers custom-made that were specific to mouse *Fak* or chicken *Fak* (Tavora et al., 2014), *Dll4* (Mm00444619_m1), *Nrarp* (Mm00482529_s1), *Hey1* (Mm00468865_m1), *Hey2* (Mm00469280_m1), *Erg* (Mm01214244_m1), *Pecam1/CD31* (Mm01246167_m1) and *Gapdh* (4352339E) (all from Applied Biosystems). The data were normalised to a *Gapdh* endogenous control to compensate for experimental variations, and also to *Pecam1* for retina tissue to compensate for alterations in EC numbers. Fold changes were calculated using the comparative cycle threshold (CT) method. For validation of adenoviral transduction in livers, quantitative real-time PCR analysis was performed using PerfeCTa SYBR Green Fastmix (95072-250, Quanta Biosciences) reagents and Erg-V5 F and Erg-V5 R primers that span the region across the Erg-V5 fusion; gene expression values were normalised to mouse *Hprt* endogenous control [primer sequences were as published previously (Sperone et al., 2011)].

### Immunoblotting analysis

Unless otherwise indicated, protein from freshly isolated primary MLECs from ECFAK^KO^ pups was extracted in RIPA lysis buffer (20-188, Millipore) supplemented with protease inhibitor cocktail set I (539131, Calbiochem). Proteins from Polyoma Middle T-immortalised endothelial cells (PMT) from ECFAK^KO^ and ECFAK^KI^ mice were extracted in lysis buffer (3% SDS, 60 mM sucrose, 65 mM Tris-HCl, pH 6.8). Samples were sonicated and protein concentration was determined with a DC Protein Assay (5000111, Bio-Rad). Lysates were heated in NuPAGE LDS sample buffer (NP0007, Thermo Fisher Scientific) at 70°C for 10 min before loading onto 10% acrylamide gels. Then, 15-30 µg of protein was analysed by SDS-PAGE. After migration, proteins were transferred to a nitrocellulose membrane (Protran BA85, 0.45 µm; GE Healthcare Life Sciences) that was blocked with 5% nonfat milk in Tris-buffered saline with 0.1% Tween-20 (TBS-T) for 1 h at room temperature and incubated overnight at 4°C with primary antibodies diluted in 4% BSA in TBS-T: FAK (Clone 77, 1:1000, 610287, BD Pharmingen), DLL4 (1:1000, #]

AF1389, R&D), Myc-tag clone 9E10 (1:1000, ab206486, Abcam), pY397-FAK (1:1000, 3283, CST), pY861-FAK (1:1000, PS1008, Invitrogen) and HSC70 (1: 5000, sc-7298, Santa Cruz Biotechnology). For ERG (1:500, ab9251, Abcam) and GAPDH (1:5000, MAB374, Millipore) immunoblotting, membranes were blocked with 3% nonfat milk in TBS for 1 h at room temperature and incubated overnight at 4°C with primary antibodies diluted in TBS-T. HRP-conjugated antibodies (1:1000, 111-035-003, 515-035-003, Jackson ImmunoResearch) were used for chemiluminescence detection with ECL western blotting (GE Healthcare) and protein levels were quantified by densitometry using ImageJ and normalised against loading controls HSC70 or GAPDH.

### Depletion of *Erg* expression *in vitro*

To silence *Erg* expression in either freshly isolated primary MLECs from ECFAK^Y861F^ pups at P6 or PMT FAK^Y861F^, ECs were seeded in MLEC media without antibiotics at 10^5^ cell/six wells the day before transfection using 20 nM siRNA against mouse ERG (SMARTpool ON-TARGETplus, L-040714-01, Dharmacon), denoted as siRNAERG in the text, and, in parallel, 20 nM siRNA ON-TARGETplus Non-targeting pool was used as negative control siRNA (D-001810-10, Dharmacon), denoted as siCtrl. Cell transfection was performed using Oligofectamine reagent (12252011, Invitrogen/Thermo Fisher Scientific) following the manufacturer's instructions. Gene expression was examined 48 h post-transfection by both quantitative real-time PCR and western blot analysis as described above.

### Mass spectrometry total and phosphoproteomic analysis

Primary mouse lung ECs preparations from ECFAK^WT^ and ECFAK^KO^ pups between P10 and P14 were prepared as described above. Briefly, 10-15 million cells were seeded in 15 cm dishes coated with 0.1% gelatin, 10 µg/ml human plasma fibronectin and 30 µg/ml collagen and incubated overnight at 37°C and 5% CO_2_. Cells were starved with serum-free, unsupplemented media (Opti-MEM I-Gibco, 31985070, Thermo Fisher Scientific) for 6 h and stimulated with either PBS (control) or VEGF (30 ng/ml) for 30 min. Total cell lysates (two biological replicates per genotype/condition) were made using SDS solubilisation buffer (2% SDS, 100 mM Tris-HCl, pH 7.5) plus 100 mM reducing buffer (DTT; D1532, Thermo Fisher Scientific). Mass spectrometry sample preparation was carried out following iFASP protocols (McDowell et al., 2013). Tandem mass tag (TMT) mass spectrometry runs were performed as described in (Dermit et al., 2020). Mass spectrometry raw data files were searched and quantified by MaxQuant software (v1.6.0.16) (Tyanova et al., 2016). The search was performed against the UniProt mouse database (2016) using the Andromeda search engine (Tyanova and Cox, 2018), with a false-discovery rate (FDR) of 0.01. The TMT 6plex search type was used, with a reporter mass tolerance of 0.01 Da. Peptide tolerance was set at 20 and 4 ppm for the first search and main search, respectively. The minimum peptide length was set to seven amino acids. The carbamidomethyl of cysteine was set as a fixed modification, whereas oxidation of methionine and protein N-terminal acetylation were set as variable modifications. For phosphoproteomics experiments, Phospho (STY) was included as an additional variable modification. Second peptides and Match between runs were also enabled. All other MaxQuant settings were kept as default. Downstream data analysis was then performed with Perseus (v1.6.2.1) ((Tyanova and Cox, 2018). First, the data were filtered for ‘Potential Contaminant’, ‘Reverse’ and ‘Only identified by site’ proteins. The data were then log2 transformed and normalised by median subtraction. A further filtration was performed based on valid values. The FAK^KO^ versus FAK^WT^ ratios were calculated for VEGF treatment and PBS (control) treatment. To identify genes that were significant outliers, Significance A was used with a 5% Benjamini–Hochberg FDR cut-off point. Category annotations were also added to each gene from the following databases: Gene Ontology biological process (GOBP), Gene Ontology cellular component (GOCC), Gene Ontology molecular function (GOMF), Kyoto Encyclopedia of Genes and Genomes (KEGG) names and UniProt keywords. Afterwards, category enrichment analysis was performed by 2D annotation enrichment (Tyanova and Cox, 2018), with the threshold set at 2% Benjamini–Hochberg FDR.

All mass spectrometry raw files and their associated MaxQuant output search results were deposited in the ProteomeXchange Consortium via the PRIDE partner repository (http://www.ebi.ac.uk/pride/archive/), under the accession number PXD030824. All mass spectrometry raw data files were searched by MaxQuant (v1.6.3.3), using a FDR of <1% for both peptide and protein identifications, calculated via a reverse database search approach. All downstream statistical analyses were performed by Perseus (v1.6.2.3). We performed Benjamini–Hochberg FDR calculations for correction of all calculated *P*-values. For calculation of outliers, an FDR cut-off of <5% was used in a two-way significance-A test. For annotation enrichment analysis, a Benjamini–Hochberg FDR cut-off of <2% was applied in the adapted Wilcoxon Mann–Whitney test.

### Cell fractionations

Primary mouse lung EC preparations from ECFAK^WT^, ECFAK^KO^, ECFAK^KIWT^, ECFAK^KD^, ECFAK^Y397F^ and ECFAK^Y861F^ pups at P6 were performed as described above. Briefly, 5 million cells were seeded in 10 cm dishes coated with 0.1% gelatin, 10 µg/ml human plasma fibronectin and 30 µg/ml collagen and incubated overnight at 37°C and 5% CO_2_. Cells were starved with serum-free, unsupplemented media (Opti-MEM I-Gibco) for 6 h and stimulated with PBS (control) or VEGF (30 ng/ml) for 30 min. Then, cells were washed in cold PBS, and lysed with Cyt buffer (10 mM Tris pH 7.5, 0.05% NP-40, 3 mM MgCl_2_, 100 mM NaCl, 1 mM EGTA containing PhosStop and cOmplete ULTRA Tablet phosphatases and protease inhibitors (4906845001, 45-5892970001, respectively, Roche). Cells were scrape-loaded into tubes, incubated for 5 min at 4°C, spun at 800 ***g*** at 4°C (5 min), and the cytosolic supernatants then collected. Cell pellets were further washed with Cyto buffer twice to remove residual cytosolic proteins. Purified nuclei were resuspended in RIPA buffer, spun at 16,000 ***g*** for 15 min, and the supernatant collected as the nuclear fraction. Alternatively, cytoplasmic and nuclear fractions were separated by using NE-PER Nuclear and Cytoplasmic Extraction Reagents (78833, Thermo Fisher Scientific) following the manufacturer's instructions. Western blotting was performed on SDS-PAGE gels. Blots were probed using the following antibodies: anti-FAK (clone 4.47) (1:1000, 05-537, Sigma-Aldrich), pY397-FAK (1:1000, 3283, CST), Lamin A/C (LMNA) (4C11, 1:1000, MAB4777, CST) and GAPDH (1:5000, MAB374, Millipore).

### Method for rapid immunoprecipitation mass spectrometry of endogenous proteins

The protocol was adapted from (Mohammed et al., 2016). Briefly, HUVECs [C2517AS (single donor), Lonza] were grown in complete media (C-22111, PromoCell). Confluent cell cultures (∼10×10^7^ per biological replicate) were starved with serum-free, unsupplemented media (Opti-MEM I-Gibco) for 6 h and stimulated with PBS (control) or VEGF (50 ng/ml) for 30 min. The media was replaced with Opti-MEM I-Gibco containing 1% formaldehyde (28908, Thermo Fisher Scientific) and crosslinked for 8 min. Crosslinking was quenched by adding glycine to a final concentration of 0.2 M. For RIME experiments, 15 μl of agarose-conjugated FAK antibody (16-173, Millipore/MERK) or 15 μg agarose-conjugated mouse IgG or Protein G agarose beads (sc-2343, Santa Cruz Biotechnology and A0919, Sigma-Aldrich, respectively) were used. For nuclear extraction, the cell pellet was resuspended in LB1 buffer (50 mM HEPES-KOH pH 7.5, 140 mM NaCl, 1 mM EDTA, 10% glycerol, 0.5% NP-40 and 0.25% Triton X-100) followed by rotation mixing for 10 min at 4°C. Then, the nuclei were pelleted and resuspended in LB2 buffer (10 mM Tris-HCL pH 8.0, 200 mM NaCl, 1 mM EDTA and 0.5 mM EGTA) and rotated at 4°C for 5 min. The samples were resuspended in LB3 buffer (10 mM Tris-HCl pH 8, 100 mM NaCl, 1mM EDTA, 0.5 mM EGTA, 0.1% Na-deoxycholate and 0.5% N-lauroylsarcosine). Chromatin/protein were sheared by sonication for 10×30 s with 30 s of rest on ice in between to produce DNA fragments of 100-1000 bp. The bead-bound antibody and chromatin/protein (300 µg total protein lysates) were incubated overnight at 4°C. The next day, the beads were washed ten times with 1 ml ice-cold RIPA buffer and twice with 500 μl 100 mM ammonium bicarbonate. The FAK nuclear protein complexes were subjected to on-bead proteolytic digestion, desalting and liquid chromatography-tandem mass spectrometry, as previously described (Turriziani et al., 2014) with the following alterations. Peptide samples were analysed using a Fusion Lumos mass spectrometer coupled to a RSLC-nano uHPLC pump (both Thermo Fisher Scientific) using a 40 min gradient (2-30% acetonitrile) over a packed emitter (40 cm, 0.075 mm ID) self-packed with 1.9 ReprosilPur AQ (NC0834952, Dr. Maisch). The Lumos mass spectrometer was operated in OT/IT mode with 120k MS resolution, rapid MS/MS IT and a cycle time of 1 s. Mass spectrometry raw data were analysed using MaxQuant 1.5 by searching against the human UniProt database using standard LFQ parameters, M(Ox) and N-terminal acetylation as variable modifications, and matching between runs. Differential expression analysis was performed using Perseus. Transcription factors associated with the mouse ERG promoter, obtained from TRRUST v2 (Transcriptional Regulatory Relationships Unravelled by Sentence-based Text mining, https://www.grnpedia.org) (Han et al., 2018) were used to seed functional protein interaction networks using QIAGEN's Ingenuity Pathway Analysis (IPA). Proteins were identified using RIME as FAK interactors under PBS (control) or VEGF-stimulated conditions. The settings used for network generation were as follows: interactions were upstream of the TF, were direct and experimentally observed. Analyses were statistically significant at *p*<0.05. Final connected networks were exported from IPA and visualised using Cytoscape 3.5.1 (Shannon et al., 2003). Data are available via ProteomeXchange with identifier PXD033447.

### Competition migration assays

Chemotaxis was studied using fluorescent colour dye prelabelled FAK^WTKI^ (green, Cell Tracker Green CMFDA, C7025, Thermo Fisher Scientific) and FAK^WTKI^, FAK^KD^, FAK^397F^ or FAK^861F^ (red, Cell Tracker Orange CMRA, C34551, Thermo Fisher Scientific) primary mouse lung ECs that were seeded at a ratio of 1:1 in Dunn chambers as in Zicha et al. (1997). Briefly, both outer and inner wells were filled with Opti-MEM I-Gibco. Cells were grown on glass coverslips overnight followed by serum starvation for at least 3 h in Opti-MEM I. Coverslips were inverted onto the Dunn Chamber and sealed on three sides with hot wax mixture (Vaseline:paraffin:beeswax, 1:1:1). The media was removed from the outer well by capillary action and was rinsed with Opti-MEM I-Gibco before filling with Opti-MEM I containing 30 ng/ml VEGF. The chamber was then sealed with wax and mounted on a fluorescence microscope ZEISS Axio100 inverted microscope equipped with a 10× objective lens and an environmental chamber that maintained 37°C and 5% CO_2_. Images were acquired by taking a frame every 10 min for 24 h using Micromanager (https://micro-manager.org). Subsequently, all cells in the acquired time-lapse sequences were tracked using the Ibidi tracking tool in ImageJ (http://rsb.info.nih.gov/ij/plugins/track/track.html).

### Competition sprouting assays

Fluorescent colour dye prelabelled as described above, FAK^WT^ (green) versus FAK^WT^ or FAK^KO^ (red) and FAK^WTKI^ (green) versus FAK^WTKI^, FAK^KD^, FAK^397F^ or FAK^861F^ (red) primary mouse lung ECs were mixed at a ratio of 1:1 (700 cells). Each cell pair set was seeded in drops of 20 μl on the lid of a 15 cm cell culture dish in MLEC medium supplemented with 0.25% methylcellulose (Sigma-Aldrich) and left overnight to form spheroids. The following day, the drops were recovered, centrifuged at 150 ***g*** with no brake for 5 min, resuspended in collagen 1 mg/ml, and left for 1 h at 37°C and 5% CO_2_. Then, EGM2 medium containing VEGF (30 ng/ml) was added to each well. The next day, the gels were fixed in PFA 4%, and analysed by confocal microscopy imaging (LSM710 microscope, 20× objective with NA 0.45; images were acquired using the multi-channel fluorescent in frame mode). The outer cell of each sprout was identified, and the ratio of red and green tip cells per total sprouts was analysed for each spheroid.

### CRISPR/Cas9 genome editing for USP9X and TRIM25

Immortalised MLECs from ECFAK^WT^, ECFAK^KO^, ECFAK^KIWT^, ECFAK^KD^, ECFAK^Y397F^ and ECFAK^Y861F^ mice were transfected with the LentiCRISPR-EGFP plasmid with inserted guide (g)RNAs for murine *Usp9x* and *Trim25*. The single guide (sg)RNAs were designed using the CRISPOR algorithm (http://crispor.tefor.net). sgRNA sequences targeting *Usp9x* (Gene ID: 22284) and *Trim25* (Gene ID: 217069) (see Table 2 for targeting sequences) were cloned into the LentiCRISPR-EGFP plasmid (Addgene ID: #75159) using the BsmBI enzyme site. MLECs were plated on cell culture plates coated with 0.1% gelatin, 10 µg/ml human plasma fibronectin and 30 µg/ml collagen and grown in MLEC media at 37°C with 5% CO_2_. Cells were centrifuged at 300 ***g*** for 5 min and resuspended in 100 μl of R1 buffer (BR5, Invitrogen), mixed with CRISPR/Cas9-EGFP plasmid DNA (10 μg) and loaded into a 100 μl Neon electroporation tip (Mpp100, Invitrogen). Electroporations were performed using 1300 mV for 20 ms with the two-pulse programme on the Neon Electroporator (Invitrogen). After electroporation, cells were left to recover in prewarmed supplemented MLEC media and cultured in six-well plates for a further 2 days.

**Table 2.**
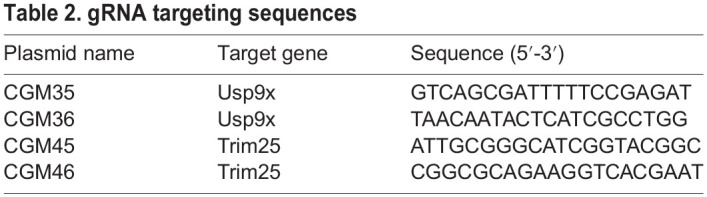
gRNA targeting sequences

The CRISPR/Cas9 KO cells were enriched by flow cytometry. Cells were washed with PBS and harvested with 200 μl of FACS buffer (1% BSA and 0.5 mM EDTA in PBS) 48 h after transfection. A 488-nm diode laser was used for the detection of EGFP. In each sample, viable singlet MLECs were gated via forward-scatter (FSC) laser and side-scatter (SSC), and EGFP-positive cells, regardless of expression levels, were sorted using a FACS AriaIII flow cytometer (BD Biosciences) at the Institute for Cancer Research Flow Cytometry and Light Microscopy Facility.

### ERG immunoprecipitation

Prior to immunoprecipitation, cells were serum starved in Opti-MEM (51985026, Gibco) for 6 h and further stimulated with murine VEGF-A (50 ng/ml; AF-450-32, PeproTech) for 30 min. Cells were washed twice with ice-cold 1× DPBS and lysed in RIPA buffer (50 mM Tris-HCl, pH 7.4, 150 mM NaCl, 1 mM EDTA, 1% NP-40, 1% sodium deoxycholic acid and 0.1% SDS) freshly supplemented with protease and phosphatase inhibitors (Cocktail I, II and III; 20-201, 539132, 524631, respectively, Millipore). Lysates were sonicated (twice for 15 s on/off at a 50% amplitude), samples were cleared by centrifugation and protein concentration determined by the Bradford protein assay (23246, Invitrogen). Equal concentrations of protein extracts were added to Protein G magnetic beads (23246, Invitrogen) coated with anti-ERG antibodies (C1, 1:1000, sc-376293, Santa Cruz Biotechnology) and rotated overnight at 4°C. Protein complexes bound to the beads were collected using a magnetic separator, washed three times with washing buffer (J628440.K3, Invitrogen), and then eluted by boiling the beads in SDS sample buffer at 95°C for 5 min. Western blotting was performed on SDS-PAGE gels. Blots were probed with an anti-ERG antibodies (1:500, ab92513, Abcam). Inputs were analysed by immunoblotting using the following antibodies: anti-USP9X (1:500, A301-351A, Cambridge Bioscience), anti-TRIM25/EFP (EPR7315) (1:1000, ab167154, Abcam), anti-ERG (EPR3864) (1:500, ab92513, Abcam), anti-ERG (C1) (1:500, sc-376293, Santa Cruz Biotechnology), anti-FAK (clone 4.47) (1:1000, 05-537, Merck Millipore) and anti-HSC70 (1:5000, sc-7298, Santa Cruz Biotechnology).

### Ubiquitin-binding domain proteins assays and proteasome inhibition

Prior to ubiquitin-binding domain protein (UBD) immunoprecipitation, cells were treated with vehicle (DMSO) or MG132 inhibitor (20 µg/ml, 474790, Millipore) for 4 h. Cells were washed twice with ice-cold 1× DPBS and lysed in Blast lysis buffer (BLR03, Cytoskeleton). Lysates were cleared by centrifugation and protein concentration determined by the Bradford protein assay (Cytoskeleton). Equal concentrations of protein extracts was incubated with UBD-coated beads incubated for 2 h at 4°C. Samples were washed three times with washing buffer (BWB02, Cytoskeleton). Proteins captured by the UBDs were released by denaturation in SDS and analysed by immunoblotting. The antibodies used included anti-ERG (EPR3864) (1:500).

### Statistical analysis

Unless otherwise indicated, all data are shown as mean±s.e.m. First, the data were subjected to Rout outlier analysis with 99% confidence, and then to a D'Agostino–Pearson normality test to analyse the distribution of the data. Parametric and nonparametric test were used depending on the outcome of that analysis. Student's *t*-test (two-tailed; unpaired) was used to compare two conditions. When more than two conditions were present, a one-way ANOVA and Šidák post-test were used for comparisons against the control sample. In the case of two groups of two conditions, a two-way ANOVA using Šidák post-test were used for comparisons between groups. All statistical analyses were generated using GraphPad Prism (GraphPad software, v8), with statistical significance indicated as: *P<0.05, **P≤0.01, ***P≤0.001, ****P≤0.0001, NS, not significant.

## Supplementary Material

Supplementary information

Reviewer comments
