# Supplementary material for: ERG activity is regulated by endothelial FAK coupling with TRIM25/USP9x in vascular patterning
Source: Development. 2022 Jul 12;149(13):dev200528. doi: 10.1242/dev.200528 (PMC9340553; doi:10.1242/dev.200528)
Supplement: Supplementary information [file develop-149-200528-s1.pdf]

Fig. S1.

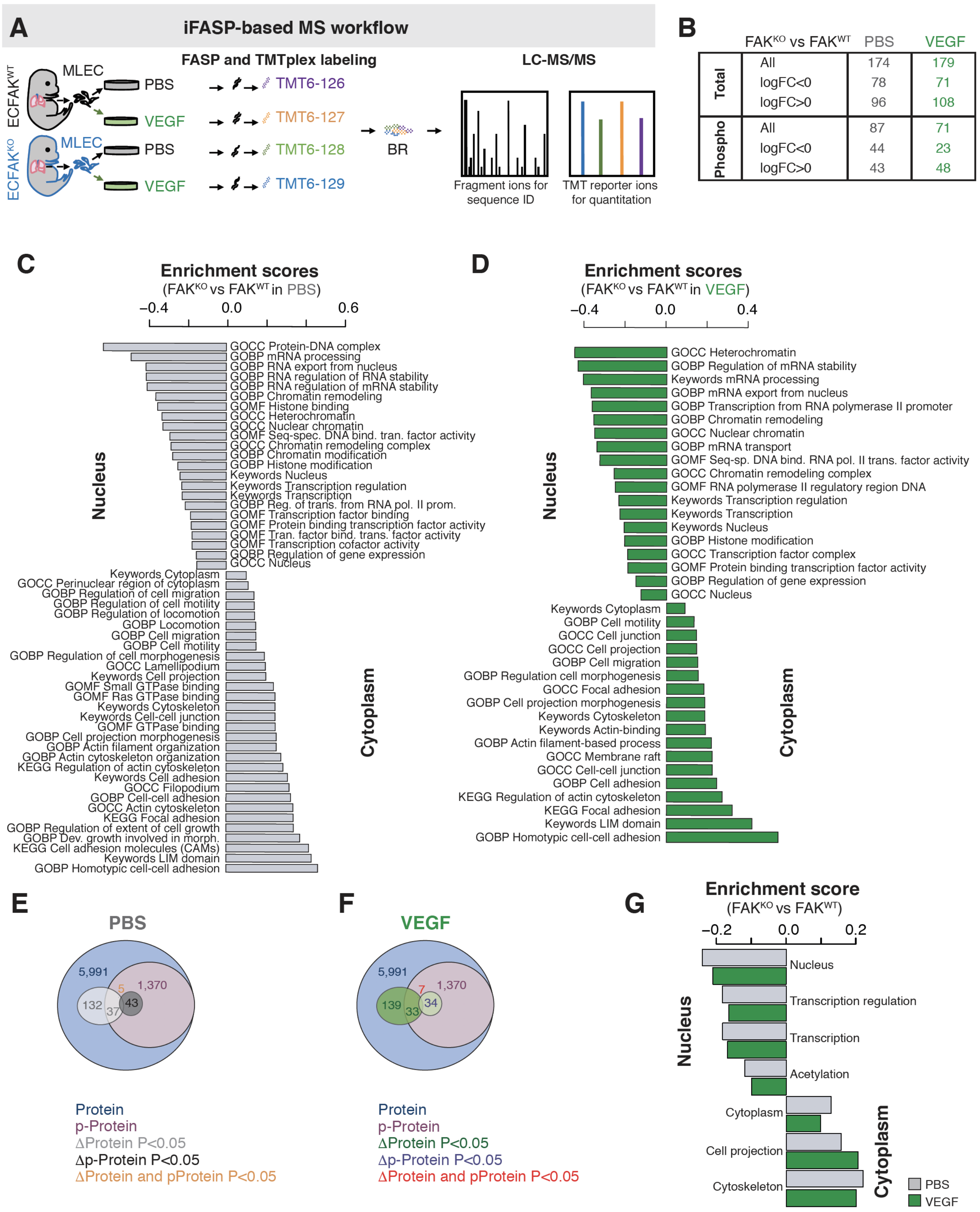

Development • Supplementary information

Fig. S1. Phospho-proteomic analysis of primary endothelial cells from ECFAKWT and ECFAKKO neonatal mice. (A) Scheme of workflow of the liquid chromatography-tandem mass spectrometry (LC-MS/MS) proteomic and phospho-proteomic analysis of lung-derived primary endothelial cells isolated ECFAKWT and ECFAKKO mice and stimulated or not with VEGF in vitro. Endothelial cell enriched populations were prepared, from lungs from ECFAKWT and ECFAKKO mice harvested at P10-P14 and expanded by sub-culturing for one passage. Two biological replicates each originated from a pool of three to four lungs of littermate mice of the same genotype. To quantify differential peptide expression levels between wild type (FAKWT) and FAK knock-out (FAKKO) primary endothelial cells PBS control (PBS) and VEGF-stimulated (VEGF), each sample was chemically labelled with isobaric tags for a combined filter-aided sample preparation (FASP) and tandem mass tag (TMT) with multiplexing approach quantification (iFASP) (McDowell et al. 2013). Peptides were then separated, sequenced, and analysed using 2D HPLC tandem high-resolution mass spectrometry (LC-MS/MS) for total phospho-proteome and proteome. **(B)** Table illustrates number of differentially expressed proteins ($p<0.05$, Benjamini-Hochberg test) and number of upregulated (logFC>0) or downregulated (logFC<0) proteins. **(C)** Proteomic profiling of primary endothelial cells from ECFAKWT and ECFAKKO neonatal mice in PBS and **(D)** VEGF stimulated conditions. Bar charts showing protein category enrichment analysis of differentially regulated proteins in FAKWT and FAKKO endothelial cells in PBS (grey bars) and VEGF (green bars) conditions. Category annotations utilized were from Gene Ontology biological process (GOBP), Gene Ontology cellular component (GOCC), Gene Ontology. molecular function (GOMF), Kyoto Encyclopedia of Genes and Genomes (KEGG) names and Uniprot Keywords databases. Positive enrichment score denotes over-representation, negative enrichment score denotes under-representation. GOBP, GOCC, GOMF, KEGG and Uniprot Keywords terms are displayed, the associated Benjamini-Hochberg corrected p values are provided in deposited datasets. Venn diagrams showing overlap of proteome and phosphor-proteome FAK-dependent datasets in **(E)** PBS and **(F)** VEGF conditions. **(G)** Bar charts showing protein category enrichment analysis of differentially regulated p-proteins in FAKWT and FAKKO endothelial cells in PBS control (grey bars) and VEGF (green bars) conditions. Category annotations utilized were from Gene Ontology biological process (GOBP), Gene Ontology cellular component (GOCC), Gene Ontology molecular function (GOMF), Kyoto Encyclopedia of Genes and Genomes (KEGG) names and Uniprot Keywords databases. Positive enrichment score denotes over-representation, negative enrichment score denotes under-representation. Selected representative Uniprot Keywords terms are displayed, the associated Benjamini-Hochberg corrected p values are provided in deposited datasets.

Development • Supplementary information

Fig. S2.

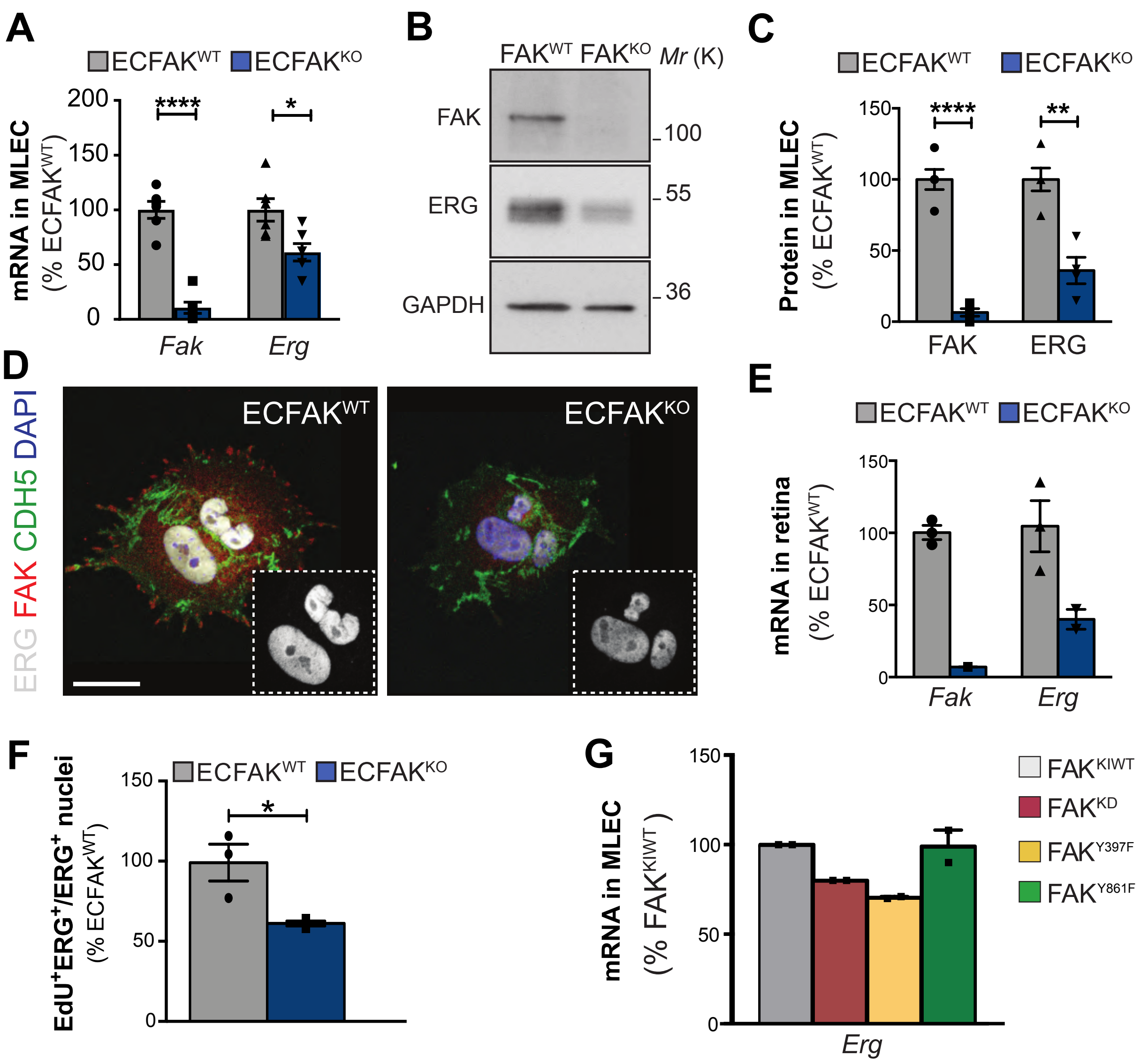

Development • Supplementary information

Fig. S2. Endothelial-cell FAK-deficiency, FAK-kinase dead and FAK-Y397F mutations downregulates Erg expression in vivo but FAK-Y861F mutation does not. (A) Transcript levels of Fak and Erg in freshly isolated primary lung ECs from P6 ECFAK^{WT} and ECFAK^{KO}. *n:* 6 independent preparations. Statistical test, Student t-test. **(B)** Western blot analysis and **(C)** densitometry quantitation of freshly isolated primary lung EC lysates show reduction of FAK and ERG expression in P6 ECFAK^{WT} and ECFAK^{KO} samples. GAPDH acts as control. *n:*2 independent EC preparations/genotype; 2-4 pups/genotype/EC preparation. Statistical test, Student t-test. **(D)** Immunofluorescence for ERG, FAK, VE-Cadherin (CDH5) and DAPI in freshly isolated primary lung ECs from ECFAKWT and ECFAKKO pups. Dashed boxes, high magnification showing ERG-stained nuclei. **(E)** qRT-PCR analysis of Fak and Erg transcripts in whole retinal tissue samples from P6 ECFAKWT and ECFAKKO mice. Transcript expressions normalized to CD31/Pecam1, relative to ECFAKWT. *n:*2-3 samples/genotype. **(F)** Retinal endothelial cell proliferation was examined by quantifying the number of double EdU- and ERG-positive cells relative to the total ERG-positive endothelial cell nuclei present in the angiogenic vascular front from ECFAK^{WT} and ECFAK^{KO} retinas. *n:*3. Student t-test used. **(G)** qRT-PCR analysis of primary endothelial cells isolated from P6 ECFAK^{KIWT}, ECFAK^{KD}, ECFAK^{Y397F} and ECFAK^{Y861F} pups. *n:*2 independent endothelial cell preparations for each ECFAKKI genotype. Each endothelial cell preparation consisted of a pool of 2-4 lungs of 4-OHT treated pups of same genotype. Note: endothelial cell lysates for mRNA isolation and western blot analysis, and endothelial cells utilized for immunofluorescence were made without culturing the cells to reflect the *in vivo* mRNA and protein levels as closely as possible. Bars, mean +/- SE. $^{**}p<0.01$, $^{***}p<0.001$, NS: not significant. Scale Bars, 10 μm.

Development • Supplementary information

Fig. S3.

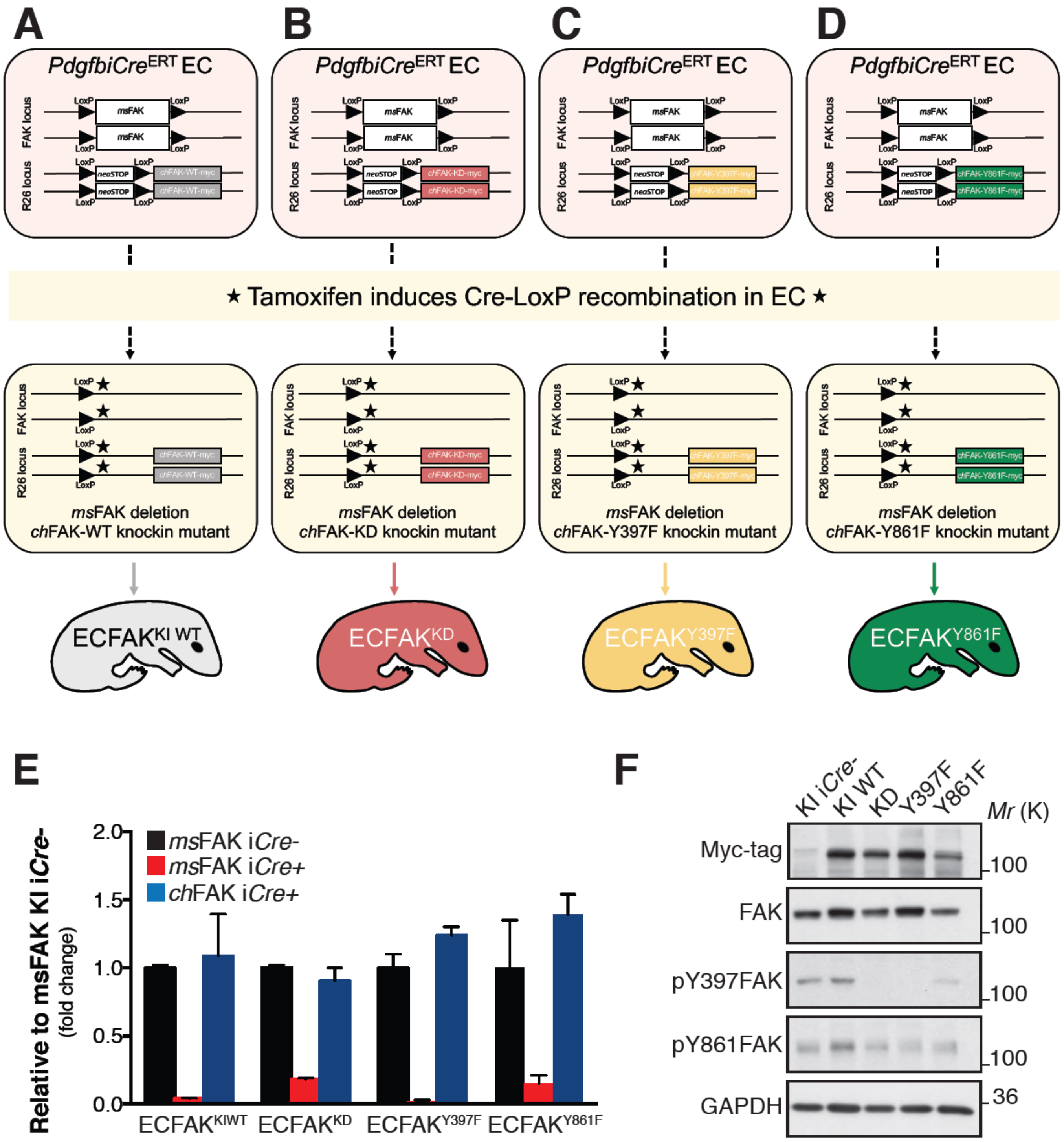

Development • Supplementary information

Fig. S3. Validation of FAK knockout/knockin system in vivo and in vitro. PdgfbiCreERT; FAK fl/fl mice (Tavora et al.2010) were intercrossed with mutant chicken FAK knockin mice (Tavora et al. 2014) to generate: PdgfbiCreERT;FAKfl/fl;R26FAKWT/WT; PdgfbiCreERT;FAKfl/fl;R26FAKKD/KD; PdgfbiCreERT;FAKfl/fl;R26FAKY397F/Y397F and PdgfbiCreERT;FAKfl/fl; R26FAKY861F/Y861F genotypes. Endogenous FAK is floxed and knockin chicken-FAK mutants were produced as homozygotes. Administration of tamoxifen induces Pdgfb-driven activation of Cre recombinase leading to simultaneous deletion of the endogenous mouse (ms) FAK gene and the neoSTOP cassette thus allowing the homozygous expression of the myc-tagged mutated chicken (ch) FAK transgene wild-type (KIWT), kinase dead (KD), non-phosphorylatable Y397 (Y387F) or non-phosphorylatable Y861 (Y861F) knockin-chicken-FAK expression specifically in endothelial cells to generate **(A)** ECFAKKIWT, **(B)** ECFAKKD **(C)** ECFAKY397F and **(D)** ECFAKY861F mice, respectively. Tamoxifen induction schedule used in this study is described in Methods. **(E)** For validation of FAK knockout/knockin strategy in vivo: primary endothelial cells were isolated from P6 pups that were treated at P1 and P2 with 4-OHT. Note: endothelial cell lysates for mRNA isolation were made without culturing the cells to reflect the in vivo mRNA levels as closely as possible. Endogenous (mouse) and chicken (knockin) mRNA FAK levels relative to Pecam1 mRNA levels - as an EC purity control - were assessed by qRT-PCR. (n:2-3 independent endothelial cell preparations for each ECFAKKI genotype. Each endothelial cell preparation consisted of a pool of 2-4 lungs of 4-OHT treated pups of same genotype. Bars, mean +/- SE (relative to endogenous msFAK mean expression in ECFAKKI iCre- corresponding control). **(F)** For validation of FAK knockout/knockin strategy in vitro: Western blot analysis of lysates from immortalized lung EC isolated from ECFAKKIWT; ECFAKKD, ECFAKY397F and ECFAKY861F mice. All iCre+ lines express Myc-tag as expected; pY397 is reduced in FAKKD and FAKY397F lysates, but less in FAKY861F lysates; pY861 is reduced in all FAK-mutants. GAPDH acts as loading control.

Development • Supplementary information

Fig.S4.

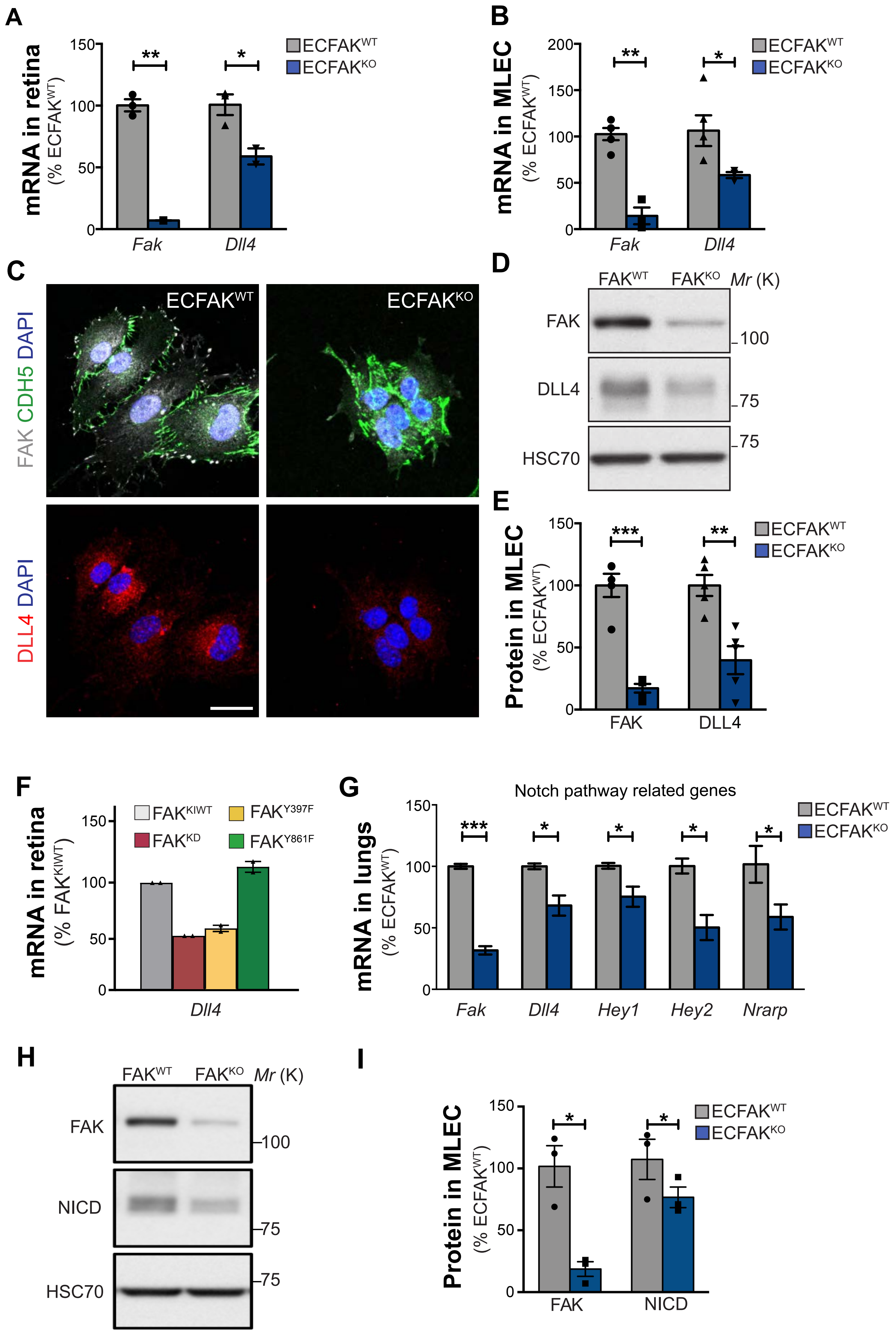

Development • Supplementary information

Fig. S4. Endothelial-cell FAK regulates DLL4 and Notch signaling effectors expression *in vivo*. (**A**) mRNA expression of *Fak* and *Dll4* levels from whole retinal tissue from P6 ECFAKWT and ECFAKKO reveals reduced expression of *Fak* and *Dll4* transcripts in ECFAKKO samples. Transcript expression is normalized to *CD31*/*Pecam1*, relative to ECFAKWT. *n*:2-3 samples/genotype; 4-6 retinas/genotype/sample. (**B**) Transcript levels of *Fak* and *Dll4* in freshly isolated primary ECs from ECFAKWT and ECFAKKO pups. *n*:3-5 independent EC preparations/experiment. Student's t-test used. (**C**) Immunofluorescence for DLL4, FAK, VE-Cadherin (CDH5) and DAPI in freshly isolated primary ECs from ECFAKWT and ECFAKKO pups. (**D**) Western blot analysis and (**E**) densitometric quantitation of FAK and DLL4 protein levels from lung derived ECs from ECFAKWT (FAKWT) and ECFAKKO (FAKKO) mice. Student's t-test used. *n:* 5 independent EC preparations/experiment. Student's t-test used. (**F**) qRT-PCR analysis of primary endothelial cells freshly isolated from P6 ECFAKKIWT, ECFAKKD, ECFAKY397F and ECFAKY861F pups. *n*:2 independent endothelial cell preparations for each ECFAKKI genotype. Each endothelial cell preparation consisted of a pool of 2-4 lungs of 4-OHT treated pups of same genotype. (**G**) mRNA expression analysis for Notch signaling effectors *Dll4*, *Hey1*, *Hey2* and *Nrarp* qRT-PCR on whole lung lysates isolated from P5 ECFAKWT and ECFAKKO mice. Deletion of endothelial FAK was induced *in vivo* by administering 4-OHT at P1 and P2. *n:2* ECFAKWT mice and *n:3* ECFAKKO littermate mice, 3 replicates per sample. Note: endothelial cell lysates for mRNA isolation and western blot analysis, and endothelial cells utilized for immunofluorescence were made without culturing the cells to reflect the *in vivo* mRNA and protein levels as closely as possible. **(H)** and **(I)** Western blot analysis of FAK and Notch intracellular domain (NICD) expression protein levels in immortalized lung-derived endothelial cells from ECFAKWT (FAKWT) and ECFAKKO (FAKKO) mice. HSC70 acts as loading control. n:3 independent EC preparations/experiment. Bars, mean +/- SE. * $p<0.05$, ** $p<0.01$, *** $p<0.001$, NS: not significant. Scale Bars, 10 μm.

Development • Supplementary information

Fig.S5.

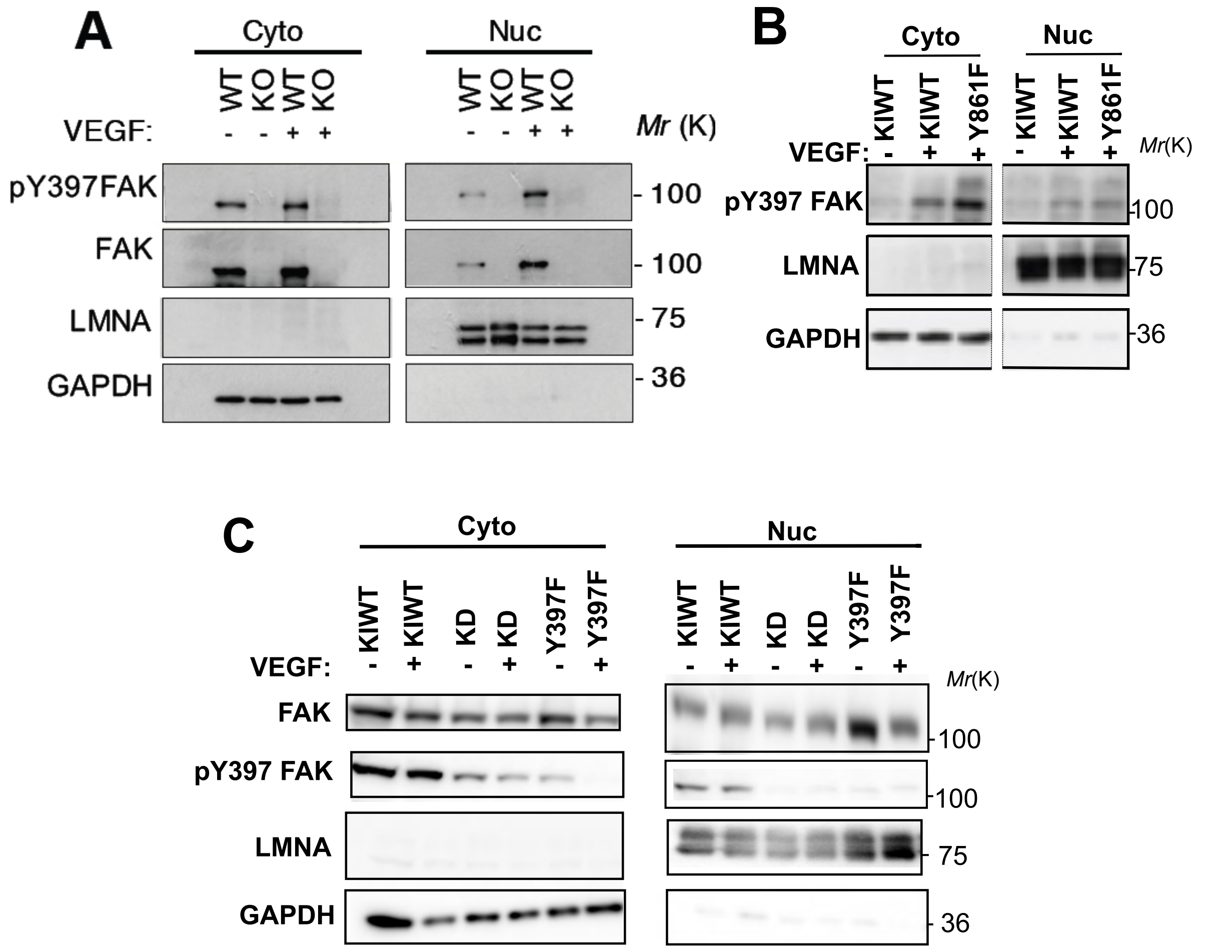

Fig. S5. Full-length wild-type and KD, Y397F and Y861F mutants of endothelial-cell FAK translocate to the nucleus in presence of VEGF. (A) Subcellular fractionation and Western blot analysis of cytoplasmic (Cyt) and nuclear (Nuc) fractions of lung-derived primary endothelial cells isolated from P6 ECFAKWT and ECFAKKO mice and stimulated with VEGF (50 ng/ml) for 30 min in vitro. and **(C)** lung-derived primary endothelial cells isolated from P6 **(B)** ECFAK^{KIWT} and ECFAK^{Y861F} and (C) ECFAK^{KIWT}, ECFAK^{Y397F} and ECFAK^{KD} and mice and stimulated with VEGF (50 ng/ml) for 30 min in vitro. n: 2 independent endothelial cell preparations for each genotype.

Development • Supplementary information

Fig.S6.

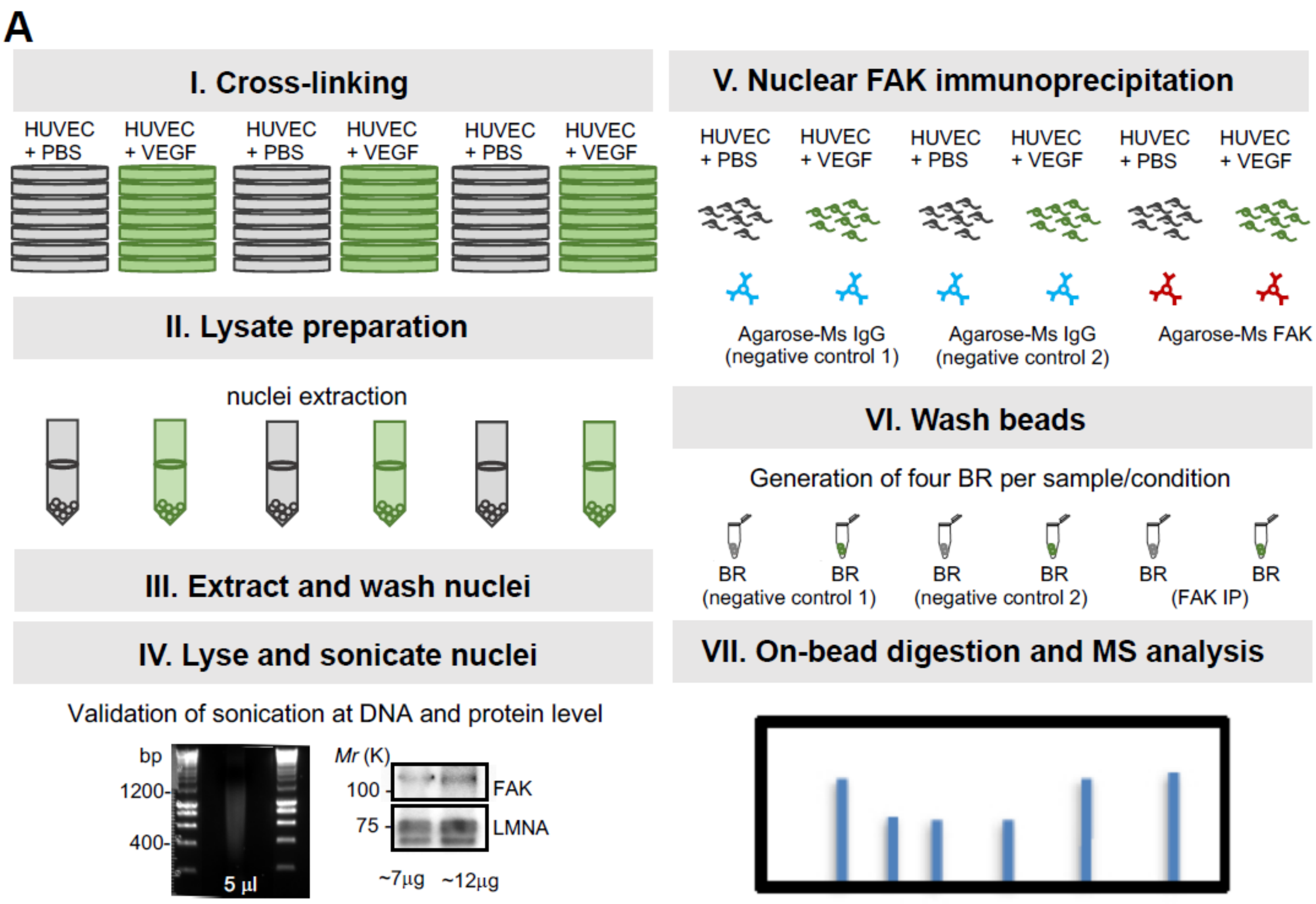

Fig. S6. Nuclear ECFAK interactome in the context of ERG transcription factor. (A) Scheme of the workflow for Rapid Immunoprecipitation Mass spectrometry of Endogenous proteins (RIME) approach used to identify nuclear endothelial-cell FAK interactome. n: 25 biological replicates of protein lysates extracted from Human Umbilical Vein Endothelial Cells (HUVEC) that were stimulated with PBS (vehicle control, n:15) or VEGF (n:10). See Materials and Methods for details.

Development • Supplementary information

Fig.S7.

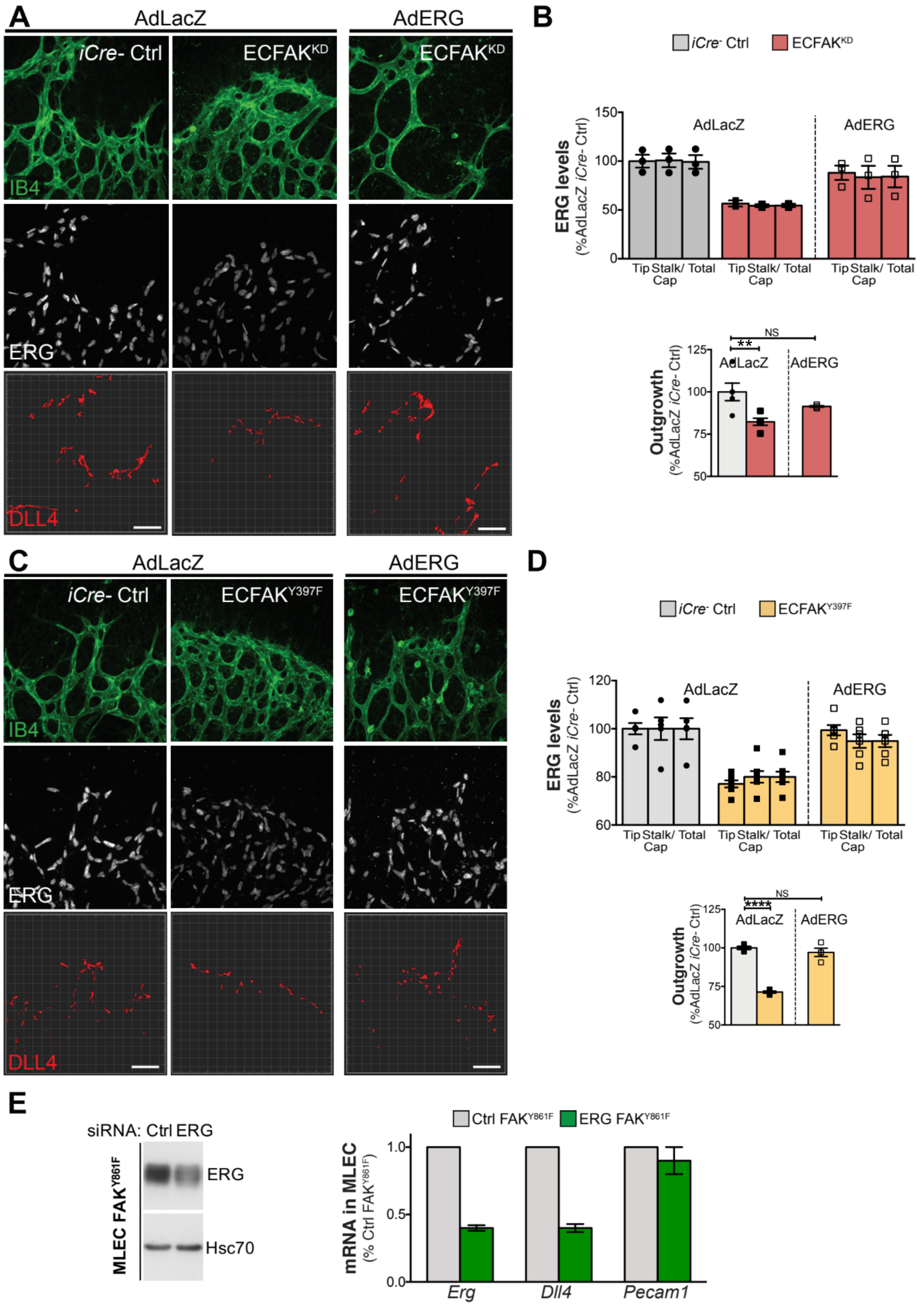

Development • Supplementary information

Fig. S7. Expression of ERG in ECFAK^{KD} and ECFAK^{Y397F} mice is sufficient to restore DLL4 expression and vascular outgrowth. (A) Confocal microscopy images of whole mount retinas immunostained for IB4, ERG and DLL4 from P6 Cre-negative controls (iCre- Ctrl) and ECFAKKD pups treated with AdLacZ or AdERG viruses. **(B)** Immunofluorescence quantitation of ERG and DLL4 expression in vivo in P6 iCre- Ctrl and ECFAKKD retinas after pup infection with AdLacZ or AdERG. Quantitation of retinal vascular outgrowth. n:3-4 pups/genotype/Ad-treatment. One-Way ANOVA test used. **(C)** Confocal microscopy images of whole-mount retinas immunostained for IB4, ERG and DLL4 from P6 iCre- Ctrl and ECFAKY397F mice treated with AdLacZ or AdERG virusesent **(D)** Immunofluorescence quantitation of ERG and DLL4 expression in P6 iCre- Ctrl and ECFAKY397F retinas after pup infection with AdLacZ or AdERG. Quantitation of retinal vascular outgrowth. *n*:4-6 pups/genotype/Ad-treatment. One-Way ANOVA test used. **(E)** Western blot analysis shows substantial reduction of ERG protein after ERG-specific siRNA transfection. Bar charts represent transcript analysis of Dll4, ERG and PECAM1 expression levels in FAKY861F ECs. n:2 independent experiments. Bars, mean + SE. $^{**}p<0.001$; $^{****}p<0.0001$ and NS, not significant. Scale bars, 50 μm.

Development • Supplementary information

Fig.S8.

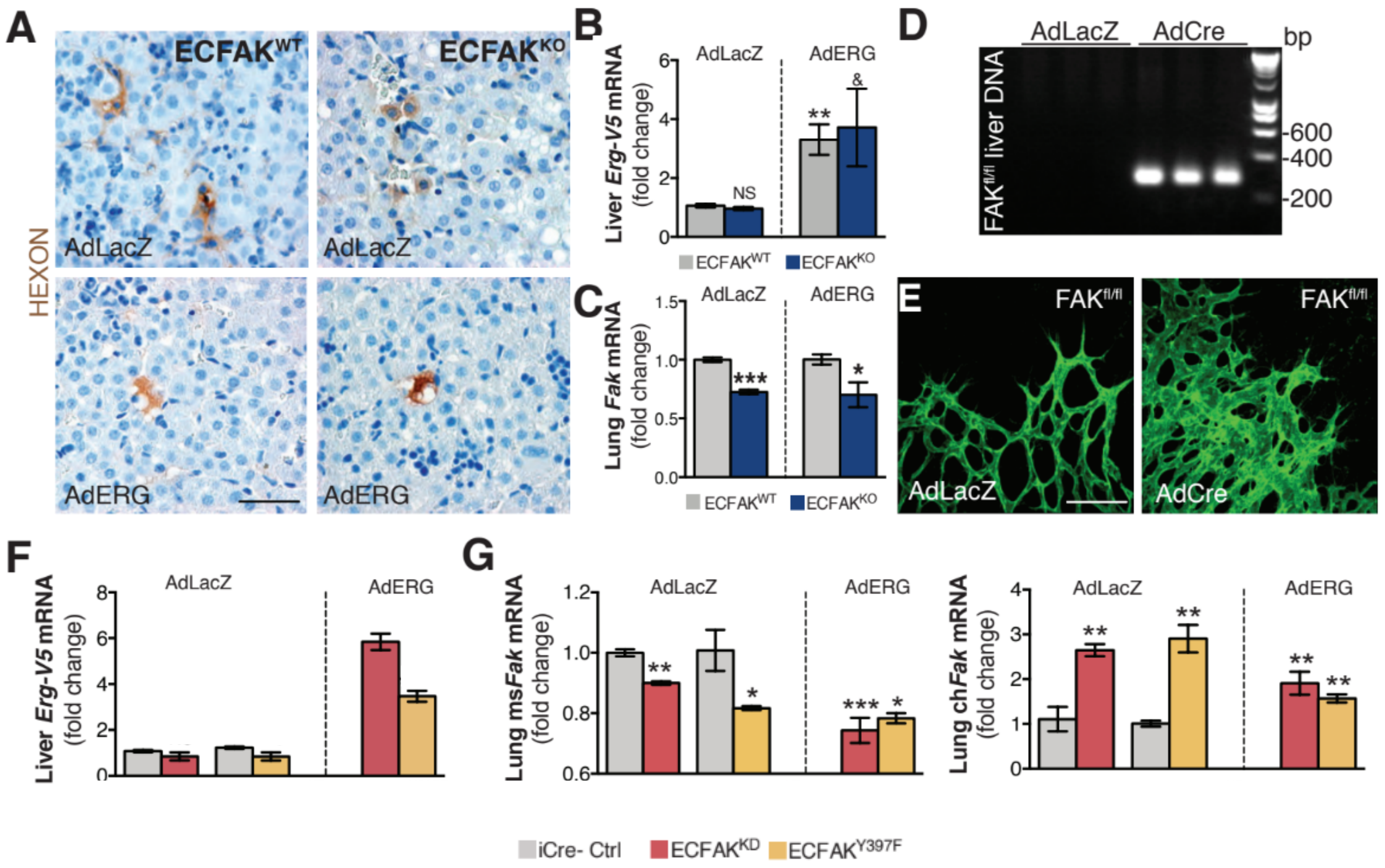

Fig. S8. Validation of the adenoviral-mediated gene expression in vivo strategy and ERG expression after Adeno-ERG transduction in vivo. **(A)** Hexon immunohistochemistry in livers from ECFAK^{KO} Adeno-treated pups. Images show chromogenic DAB immunohistochemistry in livers from ECFAKWT and ECFAK^{KO} pups stained for Hexon (brown), major coat protein in adenoviruses. Scale Bar, 100 Pm. **(B)** Quantitation of qRT-PCR for V5-Erg in livers from AdenoLacZ (AdLacZ) or AdenoERG (AdERG) infected ECFAK^{WT} and ECFAK^{KO} pups at postnatal day (P) that were subsequently administered 4-OHT tamoxifen (at P2 and P3). *n*:5 mice for ECFAK^{WT} and N=5 for ECFAK^{KO} for each Adeno-treated group. **(C)** FAK expression in ECFAK^{KO} treated with AdLacZ or AdERG. *n*:3-4 mice for ECFAK^{WT} and *n*:3 for ECFAK^{KO} for each Adeno-treated group. **(D)** PCR analysis of DNA isolated from P6 livers from AdCre and AdLacZ treated pups. Cre product: 341 bp. **(E)** Confocal microscopy images of IB4 stained retinas from FAK flox/flox AdCre and AdLacZ treated pups at P6. Scale Bar, 50 Pm. **(F)** Qrt PCR for V5-Erg in livers from AdenoLacZ- or AdenoERG-treated at postnatal day (P1) ECFAKKI mice that subsequently were administered 4-OHT tamoxifen at P2 and P3. n:2 mice for ECFAKKI iCre- and *n*:3 for ECFAKKI iCre +. **(G)** mRNA expression analysis for endogenous mouse (knockout) FAK by qRT-PCR in total lung lysates from AdenoLacZ- or AdenoERG-treated as in (F). *n*:3 mice for ECFAKKI iCre- and *n*:3 for ECFAKKI iCre+ for each Adeno-treated group. Bars, mean values +/- SE. One Way ANOVA used. * $p<0.05$, **$p<0.001$; ****$p<0.0001$ and NS, not significant.

Development • Supplementary information
