# Supplementary material for: ERG activity is regulated by endothelial FAK coupling with TRIM25/USP9x in vascular patterning
Source: Development. 2022 Jul 12;149(13):dev200528. doi: 10.1242/dev.200528 (PMC9340553; doi:10.1242/dev.200528)
Supplement: Reviewer comments [file develop-149-200528_review_history.pdf]

ERG activity is regulated by endothelial FAK coupling with TRIM25/USP9x in vascular patterning

Gabriela D'Amico, Isabelle Fernandez, Jesús Gómez-Escudero, Hyojin Kim, Eleni Maniati, Muhammad Syahmi Azman, Faraz K. Mardakheh, Bryan Serrels, Alan Serrels, Maddy Parsons, Anthony Squire, Graeme M. Birdsey, Anna M. Randi, Alfonso Bolado-Carrancio, Rathi Gangeswaran, Louise E. Reynolds, Natalia Bodrug, Yaohe Wang, Jun Wang, Pascal Meier and Kairbaan M. Hodivala-Dilke

DOI: 10.1242/dev.200528

Editor: Liz Robertson

Review timeline

Original submission:	19 August 2020
Editorial decision:	8 October 2020
Resubmission:	20 January 2022
Editorial decision:	4 April 2022
First revision received:	26 April 2022
Accepted:	29 April 2022

Original submission

Decision letter

MS ID#: DEVELOP/2020/196170

MS TITLE: ERG activity is regulated by endothelial FAK coupling with TRIM25/USP9x in vascular patterning

AUTHORS: Gabriela D'Amico, Isabelle Fernandez, Jesus Escudero, Hyojin Kim, Eleni Maniati, Muhammad Syahmi Azman, Faraz Mardakheh, Bryan Serrels, Alan Serrels, Maddy Parsons, Anthony Squire, Graeme M Birdsey, Anna M Randi, Peter Hollenhorst, Rathi Gangeswaran, Louise E Reynolds, Natalia Bodrug, Yaohe Wang, Jun Wang, Pascal Meier, and Kairbaan M Hodivala-Dilke

Dear Dr. D'Amico,

I have now received all the referees' reports on the above manuscript, and have reached a decision. The referees' comments are appended below, or you can access them online: please go to BenchPressand click on the 'Manuscripts with Decisions' queue in the Author Area.

As you will see, the referees express considerable interest in your work, but have some significant criticisms and recommend a substantial revision of your manuscript before we can consider publication. If you are able to revise the manuscript along the lines suggested, which may involve further experiments, I will be happy receive a revised version of the manuscript. Your revised paper will be re-reviewed by one or more of the original referees, and acceptance of your manuscript will depend on your addressing satisfactorily the reviewers' major concerns. Please also note that Development will normally permit only one round of major revision.

We are aware that you may be experiencing disruption to the normal running of your lab that make experimental revisions challenging. If it would be helpful, we encourage you to contact us to discuss your revision in greater detail. Please send us a point-by-point response indicating where you are able to address concerns raised (either experimentally or by changes to the text) and

© 2022. Published by The Company of Biologists under the terms of the Creative Commons Attribution License (https://creativecommons.org/licenses/by/4.0/).

where you will not be able to do so within the normal timeframe of a revision. We will then provide further guidance. Please also note that we are happy to extend revision timeframes as necessary. Please attend to all of the reviewers' comments and ensure that you clearly highlight all changes made in the revised manuscript. Please avoid using 'Tracked changes' in Word files as these are lost in PDF conversion. I should be grateful if you would also provide a point-by-point response detailing how you have dealt with the points raised by the reviewers in the 'Response to Reviewers' box. If you do not agree with any of their criticisms or suggestions please explain clearly why this is so.

Liz Robertson
Handling Editor

Development

Reviewer 1

Advance summary and potential significance to field

The article entitled "ERG activity is regulated by endothelial FAK coupling with TRIM25/USP9x in vascular patterning" investigates the mechanism by which FAK (or PTK2) regulates vascular patterning in the mouse retina in great depth. More specifically, the authors show that nuclear FAK in vascular endothelial cells interacts with the deubiquitinase USP9x and ubiquitin ligase TRIM25 to promote ERG and DLL4 expression levels. The study therefore proposes a completely novel mode of how the key angiogenic factors ERG, FAK and DLL4 functionally interact with each other by providing evidence for an FAK- mediated control of ERG phosphorylation and ubiquitination events to govern retinal angiogenesis. The report includes a remarkably high number of diverse state-of-the-art methods, including endothelial cell- specific inducible gene deletion mouse models, knock-in mouse models, LC-MS proteomic analyses, retinal sprout analyses, endothelial competition assays, IP and interactome methods, Crispr/cas9 knockouts in endothelial cells as well as dynamic analyses of endothelial cell migration.

Comments for the author

In its current state, the manuscript looks as if it has already been intensively peer-reviewed by a top journal and therefore is loaded with controls and many sophisticated mechanistic details. According to this referee, the manuscript could be published as is, and only minor points need to be addressed using textual changes:
1. The abstract reads a bit complicated and the authors might consider writing it in a bit more punchy way with more emphasis on the proposed mechanism than applied methods (which are totally impressive, but do not represent the "take-home-message").
2. In Figure 1, this referee searched for FAK as being affected and only realized after looking through this Figure that PTK2 is the same as FAK. The authors therefore need to introduce PTK2 as synonym in the introduction.
3. The authors compared ECFAK KO mice with ECFAK WT mice. Unfortunately, the latter do not contain the Cre gene that can sometimes affect sprouting angiogenesis. Normally, this referee would ask for a Cre control, but since the authors continue to use knock-in approaches, strongly confirming (and expanding) their findings made in these mice, they should just disclose in the text the lack of Cre gene in their ECFAK WT mice.
4. The authors write about their knock-in approach, but should mention somewhere in the text that this approach might represent a comparison of over-expressed WT FAK versus mutant FAKs (since these FAK genes are under ROSA26 control).

Reviewer 2

Advance summary and potential significance to field

This manuscript would help to understand how important signaling molecules and transcriptional factors coordinate to regulate angiogenesis.

© 2022. Published by The Company of Biologists under the terms of the Creative Commons Attribution License (https://creativecommons.org/licenses/by/4.0/).

Comments for the author

The manuscript by D'Amico and Fernandez et al., reports on the function of FAK as a regulator of ERG/DLL4 transcriptional pathway in vascular patterning by coupling TRIM25/USP9 complex to ERG expression, phosphorylation and activity. Specifically, authors investigate the role of the nuclear functions of FAK in endothelial cells, by combining cellular and mouse models with imaging and biochemistry approaches. The strength of this manuscript relies on the beautiful in vivo models used, where the experiments were carefully designed, including all key controls. This is in contrast with the in vitro approach which is much less conclusive and often lacks rationale. The authors need to clarify several points as described below.

In this manuscript, the authors investigate three putative levels of regulation of ERG by FAK (Transcription, Ubiquitination, and Phosphorylation). However, there is no real connection between them, or at least the data provided do not support this. This should be clarified as this tremendously confuses the reader. For instance, the authors demonstrate the FAK regulates ERG transcription expression (Fig1, Fig2) and that this affects DLL4 expression. Then, they looked for FAK nuclear binding partners and ERG-associated transcription factors to explain the molecular mechanism of regulation, but instead of focusing in how FAK regulates transcription, they select USP9 and TRIM25 as FAK-binding partners to study ERG protein regulation (previously published that regulate ERG stability (stabilization and degradation). While this is interesting as may suggest a multifactorial way of ERG regulation by FAK, it is unclear how is all connected. So how does (exactly) FAKs regulate transcriptionally ERG expression? Then the concept of phosphorylation is brought into the manuscript, but with not clear rationale/connection with the other two elements. Do posttraductional modifications affect mRNA levels? Does FAK have scaffolding function by bringing USP9 or TRIM25 close to ERG? Does FAK phosphorylate ER directly? How does phospho levels of ERG connect to transcription or posttranscriptional modifications done by USP or TRIM25? The authors focus on the regulation of ERG at its phosphorylation state and its posttranslational modifications. However, it is not clear how this regulates ERG levels, because total ERG levels shown in Fig 4C and D-right panel are different. While in 4C TRIM25 deletion affects ERG levels between FAKWT and FAKKO in 4D right panel there is not difference. Also, I miss the FAKKO gControl condition without treatment in the Fig4D. On the other hand, no difference is observed in the levels of ERG (poly)ubiquitination in the presence of proteasome inhibitor MG132 between FAKWT and FAKKO ECs. Does this suggest that ERG polyubiquitination is independent of FAK? How authors connect FAK - USP9/TRIM25 in the regulations of ERG ubiquitination? Overall, Figure. 4 is very immature in its current form. Data often do not support the conclusions reached by the authors. As this is an important aspect of this manuscript, authors should clarify/ simplify the link between FAK/ERG. A way to do that would be to focus on one aspect and leave the rest for a follow up study.

Another unresolved questions from this study is whether FAK regulates ERG expression upon VEGF stimulation only? It is not clear why author address the transcriptomic program induced by FAK depletion in endothelial cells under VEGF conditions. Similarly, why do authors choose to study ERG as a critical FAK downstream effector? In Fig1A-C they showed 86 commonly differentially expressed between WT and KO in PBS and VEGF. They select ERG because is downregulated under FAK KO stimulated with VEGF. However, it is not clear whether ERG decrease only under VEGF stimulation or also under PBS or whether VEGF stimulates ERG expression in WT cells. This should be clarified. At the end of the day, authors claim that ERG expression is sustained by FAK, the question here is whether this is under basal, under VEGF, or both. The key message at this point is confusing. It would be nice to include a western blot for ERG of WT and FAKKO EC with and without VEGF stimulation.

The authors demonstrate that FAK can be translocated to the nucleus independent of its phosphorylated state, however, they don't explore whether its phosphorylated state determine the total level of FAK that translocate or whether this could explain the VEGF-depend response (translocation). While on FigS5A it is clear that VEGF promotes FAKs translocation, it is not clear whether or how its level of phosphorylation affects its translocation. FAK KD translocation is not sensitive to VEGF and they don't show how Y397F or Y861F affect to FAK translocation. This is important to rule out that this is the reason why there is a FAK- dependent decrease in ERG expression/activation (lack of total FAK decrease ERG level, does mutant for Y397 avoid increased levels of nuclear FAK under VEGF stimulation?).

© 2022. Published by The Company of Biologists under the terms of the Creative Commons Attribution License (https://creativecommons.org/licenses/by/4.0/).

There are many experiments that have been performed 1 or 2 times. This need to be revised. Similarly, it is not clear how they have performed the statistical test when they only have an n of 2. Also, in these cases the dots in the graph are missing. In the graphs with more than two conditions they need to revise the statistical test performed. T test for separate groups is not appropriate for more than two groups (use Anova).
Authors need to explain which type of immortalized endothelial cells they use for the Crispr experiments.

Some consistency in the style of the legend is recommended.

I miss this reference in the intro or discussion.
https://www.ncbi.nlm.nih.gov/pmc/articles/PMC5803223/pdf/41598_2018_Article_20930.pdf

---

First revision

Author response to reviewers' comments

Reviewer 1:

Advance Summary and Potential Significance to Field:

The article entitled "ERG activity is regulated by endothelial FAK coupling with TRIM25/USP9x in vascular patterning" investigates the mechanism by which FAK (or PTK2) regulates vascular patterning in the mouse retina in great depth. More specifically, the authors show that nuclear FAK in vascular endothelial cells interacts with the deubiquitinase USP9x and ubiquitin ligase TRIM25 to promote ERG and DLL4 expression levels. The study therefore proposes a completely novel mode of how the key angiogenic factors ERG, FAK and DLL4 functionally interact with each other by providing evidence for an FAK mediated control of ERG phosphorylation and ubiquitination events to govern retinal angiogenesis. The report includes a remarkably high number of diverse state-of-the-art methods, including endothelial cell specific inducible gene deletion mouse models, knock-in mouse models, LC-MS proteomic analyses, retinal sprout analyses, endothelial competition assays, IP and interactome methods, Crispr/cas9 knockouts in endothelial cells as well as dynamic analyses of endothelial cell migration.

Comments for the Author:
In its current state, the manuscript looks as if it has already been intensively peer-reviewed by a top journal and therefore is loaded with controls and many sophisticated mechanistic details. According to this referee, the manuscript could be published as is, and only minor points need to be addressed using textual changes:

We thank the reviewer for highlighting the novelty and significance of our work. We are also pleased that the manuscript could be published as is.

1. The abstract reads a bit complicated and the authors might consider writing it in a bit more punchy way with more emphasis on the proposed mechanism than applied methods (which are totally impressive, but do not represent the "take-home-message").

We thank the reviewer for this comment and have rewritten the abstract to be more focused on the phenotype and proposed mechanism than in the methods.

2. In Figure 1, this referee searched for FAK as being affected and only realized after looking through this Figure that PTK2 is the same as FAK. The authors therefore need to introduce PTK2 as synonym in the introduction.

We apologise for this omission, we have now introduced PTK2 as synonym of FAK in Figure 1B and 1D, and in the introduction of our revised MS (see in Introduction, page 4, line 101):

© 2022. Published by The Company of Biologists under the terms of the Creative Commons Attribution License (https://creativecommons.org/licenses/by/4.0/).

"Focal adhesion kinase (FAK), also known as PTK2 protein tyrosine kinase (PTK2), is a non-receptor…"

3. The authors compared ECFAK KO mice with ECFAK WT mice. Unfortunately, the latter do not contain the Cre gene that can sometimes affect sprouting angiogenesis. Normally, this referee would ask for a Cre control, but since the authors continue to use knock-in approaches, strongly confirming (and expanding) their findings made in these mice, they should just disclose in the text the lack of Cre gene in their ECFAK WT mice.

Thank you for this comment. We have now clarified in the text that the ECFAK WT mice are tamoxifen treated Cre-negative. Please see table1 in page 15, line 360.

4. The authors write about their knock-in approach, but should mention somewhere in the text that this approach might represent a comparison of over-expressed WT FAK versus mutant FAKs (since these FAK genes are under ROSA26 control).

Although FAK-expression is under ROSA26 promoter control our data indicate that inserted-FAK is not actually over expressed. Please see previously published data where western blot analysis shows no significant difference in total FAK expression between endogenous mouse FAK in ECs isolated from WT mice and knocked-in FAK expression under the ROSA26 promoter, (Tavora et al., Genesis 2014, Fig 3).
Additionally in the current manuscript we show that chicken FAK RNA and protein levels are similar to endogenous mouse FAK in ECs in which Cre has not been activated (Please see Supp fig 3E and F).

Reviewer 2:

Advance Summary and Potential Significance to Field:
This manuscript would help to understand how important signaling molecules and transcriptional factors coordinate to regulate angiogenesis.

Comments for the Author:
The manuscript by D'Amico and Fernandez et al., reports on the function of FAK as a regulator of ERG/DLL4 transcriptional pathway in vascular patterning by coupling TRIM25/USP9 complex to ERG expression, phosphorylation and activity. Specifically, authors investigate the role of the nuclear functions of FAK in endothelial cells, by combining cellular and mouse models with imaging and biochemistry approaches. The strength of this manuscript relies on the beautiful in vivo models used, where the experiments were carefully designed, including all key controls.

We thank the reviewer for highlighting that our work is of interest.

This is in contrast with the in vitro approach which is much less conclusive and often lacks rationale. The authors need to clarify several points as described below:

(1) In this manuscript, the authors investigate three putative levels of regulation of ERG by FAK (Transcription, Ubiquitination, and Phosphorylation). However, there is no real connection between them, or at least the data provided do not support this. This should be clarified as this tremendously confuses the reader. For instance, the authors demonstrate the FAK regulates ERG transcription expression (Fig1, Fig2) and that this affects DLL4 expression. Then, they looked for FAK nuclear binding partners and ERG-associated transcription factors to explain the molecular mechanism of regulation, but instead of focusing in how FAK regulates transcription, they select USP9 and TRIM25 as FAK-binding partners to study ERG protein regulation (previously published that regulate ERG stability (stabilization and degradation). While this is interesting as may suggest a multifactorial way of ERG regulation by FAK, it is unclear how is all connected.

We respectfully suggest that reviewer's concerns regarding the term 'FAK affects ERG transcription expression' are a misunderstanding. To clarify, in Fig 1, we have not examined the ERG transcription regulation, but rather these data are proteomics analysis, thus they address FAK-

© 2022. Published by The Company of Biologists under the terms of the Creative Commons Attribution License (https://creativecommons.org/licenses/by/4.0/).

regulation of ERG protein levels in ECs. We have reclarified this in the revised results text for Fig.1 and Fig S1 A and B. Please see page 6 lines 140-143.

Skipping to one of the final comments from this reviewer:

(11) Overall, Figure. 4 is very immature in its current form. Data often do not support the conclusions reached by the authors. As this is an important aspect of this manuscript, authors should clarify/ simplify the link between FAK/ERG. A way to do that would be to focus on one aspect and leave the rest for a follow up study.

We agree that focusing on different levels of ERG regulation may be complicated and so we have taken the decision to follow this reviewer's advice (point 11 of original review) and focus our work on the role of FAK in ERG ubiquitinylation and have amended the in vitro work in Fig 4 accordingly.

(2) So how does (exactly) FAKs regulate transcriptionally ERG expression?

(3) Then the concept of phosphorylation is brought into the manuscript, but with not clear rationale/connection with the other two elements. Do posttraductional modifications affect mRNA levels?

How ERG is transcriptional regulated by FAK specifically in endothelial cells is unclear at present and beyond the scope of the present study. To discuss this point in this response to reviewers' comments, literature searches suggest that ERG expression can be self-regulated in a positive loop, thus a decrease on protein levels by post-translational regulations could affect transcriptional expression of ERG (Mani, et al 2011; Thoms, et al 2011). Interestingly FAK can regulate GATA-4 expression (Jeong et al, 2019) and a mutation in GATA-4 can regulate ERG expression in cardiogenesis (Ang et al, 2016). Additionally, ERG serine phosphorylation status has been related to ERG expression levels (Selvaraj et al., 2015; Kedage et al., 2017; Fish et al., 2017) and we will further investigate this aspect of the mechanism in a future study.

(4) Does FAK phosphorylate ERG directly? (5) How does phospho levels of ERG connect to transcription or posttranscriptional modifications done by USP or TRIM25?

Since FAK is a non-receptor tyrosine kinase ERG serine phosphorylation it is unlikely to be targeted directly by FAK. Although indirect effects have been implicated (Wrobel et al, 2020) how USP9X or TRIM25 directly affects phosphorylation levels of ERG is unknown. These questions will be followed up in a separate manuscript to avoid confusing the message of the current revised manuscript which focuses on the ubiquitinylation of ERG controlled by FAK-USP9X/TRIM25.

6. Does FAK have scaffolding function by bringing USP9 or TRIM25 close to ERG?

In the original submission Rapid Immunoprecipitation spectrometry of Endogenous proteins (RIME) established novel nuclear FAK specific interactors in endothelial cells (ECs) including USP9x and TRIM25 (Please see Fig. 4A and B, and Fig S6A). Given the downregulation of ERG protein in FAKKO ECs (Fig 1D), in silico analysis of the ERG-transcription factor seeding network in the nuclear FAK-interactome was investigated. This identified USP9x and TRIM25 as the most highly significant nuclear FAK-interactome molecules that have reported protein-protein binding with ERG (Fig4 A and B). Thus, we focused our efforts on the FAK-USP9x/TRIM25-ERG network. In preliminary experiments ERG immunoprecipitation followed by western blotting for USP9x or RIM25 shows no significant differences in the expression levels between WT vs FAKKO ECs (data not shown). This suggests that the differential effect of USP9x and TRIM25 on ERG expression levels in the presence or absence of FAK is not likely due to a scaffolding function of FAK in bringing USP9x and TRIM25 close to ERG. The molecular basis of the interaction of FAK with USP9x and TRIM25 is a large body of work that will be followed up in subsequent studies. Furthermore, as ERG expression is also reduced in EC-FAKY397F and EC-FAKKD mice, it is more likely that the function of FAK is related within its activity and phosphorylated status. Thus, whether FAK has scaffolding functions by bringing USP9x or TRIM25 close to ERG is of interest but something that we would like to further investigate in depth in a future study.

© 2022. Published by The Company of Biologists under the terms of the Creative Commons Attribution License (https://creativecommons.org/licenses/by/4.0/).

7. The authors focus on the regulation of ERG at its phosphorylation state and its posttranslational modifications. However, it is not clear how this regulates ERG levels, because total ERG levels shown in Fig 4C and D-right panel are different. While in 4C TRIM25 deletion affects ERG levels between FAKWT and FAKKO in 4D right panel there is not difference.
In the resubmitted paper we have significantly revised this figure, and as this reviewer suggests, we have focused on the ubiquitinylation of ERG controlled by FAK instead of ERG phosphorylation.

The regulation of ERG protein is as follows. Using RIME analysis we show that FAK interacts directly with USP9x and TRIM25 (Figure 4A). Using the TRRUST v2 database interrogation ERG_TF associated seeding identifies protein-protein interactions of ERG with USP9x and TRIM25. We have revised and repeated the experiments using CRISPR-Cas9 mediated depletion of USP9x and TRIM25. We have confirmed a down regulation of ERG levels in FAKKO ECs, regulated by USP9x and TRIM25 (Please see revised Fig 4C).

To confirm that proteasomal degradation is implicated in the regulation of ERG expression we have: (1) treated FAKWT and FAKKO ECs with the proteasome inhibitor MG132; (2) immunoprecipitated ubiquitinated proteins; and (3) immunoblotted for ERG. A significant increase of ubiquitinated ERG in the FAKKO EC compared with FAKWT ECs was observed (Please see revised Fig 4D).

Lastly, we have analysed if total ERG levels could be rescued by proteasomal degradation inhibition. Treatment with MG132 rescue the levels of ERG in FAKKO cells to a similar level of FAKWT cells, highlighting the role of ERG degradation in the context of FAK expression in ECs. (Please see revised Fig. 4E).

8. Also, I miss the FAKKO gControl condition without treatment in the Fig4D.
9. On the other hand, no difference is observed in the levels of ERG (poly)ubiquitination in the presence of proteasome inhibitor MG132 between FAKWT and FAKKO ECs. Does this suggest that ERG polyubiquitination is independent of FAK? How authors connect FAK-USP9/TRIM25 in the regulations of ERG ubiquitination?

The data previously included in Fig 4D has now been replaced with additional data focusing on ERG degradation. We show that ERG (poly)ubiquitination is dependent on EC FAK expression. ERG ubiquitinylation is higher in FAKKO ECs compared to FAKWT ECs (Please see revised Fig 4D).

Additionally, treatment with the proteasome inhibitor MG132 rescues the expression of ERG in FAKKO ECs (Please see revised Fig 4E). Our results suggest that USP9x de-ubiquitinase promotes stabilisation of ERG protein expression in ECs. In contrast, TRIM25 ubiquitin ligase promotes degradation of ERG protein (Please see revised Fig. 4C).

10. The authors demonstrate that FAK can be translocated to the nucleus independent of its phosphorylated state, however, they don't explore whether its phosphorylated state determine the total level of FAK that translocate or whether this could explain the VEGF depend response (translocation). While on FigS5A it is clear that VEGF promotes FAKs translocation, it is not clear whether or how its level of phosphorylation affects its translocation. FAK KD translocation is not sensitive to VEGF and they don't show how Y397F or Y861F affect to FAK translocation. This is important to rule out that this is the reason why there is a FAK dependent decrease in ERG expression/activation (lack of total FAK decrease ERG level, does mutant for Y397 avoid increased levels of nuclear FAK under VEGF stimulation?).

We thank the reviewer for this observation. In the revised manuscript we show that mutant Y397F-FAK can translocate to the nuclei in basal and VEGF-stimulated conditions (Please see new data in Fig S5 C).

12. There are many experiments that have been performed 1 or 2 times. This need to be revised. Similarly, it is not clear how they have performed the statistical test when they only have an n of 2. Also, in these cases the dots in the graph are missing. In the graphs with more than two conditions they need to revise the statistical test performed. T test for separate groups is not appropriate for more than two groups (use Anova).

© 2022. Published by The Company of Biologists under the terms of the Creative Commons Attribution License (https://creativecommons.org/licenses/by/4.0/).

We have increased the number of replicates for individual experiments in the manuscript. All n-numbers are given in respective figure legends. We would like to emphasise that we have performed statistical analysis only with at least 3 replicates. We have revised and redone the statistical analysis, using the appropriate method for each case.

In addition, detailed explanation of the statistical analyses according to the data type and numbers of groups is shown in Material and Methods (Please see revised statistical Methods, page 31).

13. Authors need to explain which type of immortalized endothelial cells they use for the Crispr experiments.
We have immortalized mouse lung endothelial cells, 'immortalized MLEC'. Please see original submission in Material and Methods (Please see page 19 line 451).

14. Some consistency in the style of the legend is recommended.

We thank the reviewer for this comment, and we would like to confirm that in our revised we have written the legends in a consistent style, adhering to Development guidelines.

15. I miss this reference in the intro or discussion.
https://www.ncbi.nlm.nih.gov/pmc/articles/PMC5803223/pdf/41598_2018_Article_20930.pdf

We apologize for this omission, this reference is now included in Introduction, (page 5, line 114) in our revised manuscript.

Bibliography

FISH, J. E., GUTIERREZ, M. C., DANG, L. T., KHYZHA, N., CHEN, Z., VEITCH, S., CHENG, H. S., KHOR, M., ANTOUNIANS, L., NJOCK, M. S., BOUDREAU, E., HERMAN, A. M., RHYNER, A. M., RUIZ, O. E., EISENHOFFER, G. T., MEDINA-RIVERA, A., WILSON, M. D., & WYTHE, J. D. 2017. Dynamic regulation of VEGF-inducible genes by an ERK/ERG/p300 transcriptional network. Development (Cambridge). https://doi.org/10.1242/dev.146050
JEONG, K., KIM, J. H., MURPHY, J. M., PARK, H., KIM, S. J., RODRIGUEZ, Y. A. R., KONG, H., CHOI, C., GUAN, J. L., TAYLOR, J. M., LINCOLN, T. M., GERTHOFFER, W. T., KIM, J. S., AHN, E. Y. E., SCHLAEPFER, D. D., & LIM, S. T. S. 2019. Nuclear focal adhesion kinase controls vascular smooth muscle cell proliferation and neointimal hyperplasia through GATA4-mediated cyclin D1 transcription. Circulation Research. https://doi.org/10.1161/CIRCRESAHA.118.314344
KEDAGE, V., STRITTMATTER, B. G., DAUSINAS, P. B., & HOLLENHORST, P. C. 2017. Phosphorylation of the oncogenic transcription factor ERG in prostate cells dissociates polycomb repressive complex 2, allowing target gene activation. Journal of Biological Chemistry. https://doi.org/10.1074/jbc.M117.796458
MANI, R. S., IYER, M. K., CAO, Q., BRENNER, J. C., WANG, L., GHOSH, A., CAO, X., LONIGRO, R. J., TOMLINS, S. A., VARAMBALLY, S., & CHINNAIYAN, A. M. 2011. TMPRSS2-ERG-mediated feed-forward regulation of wild-type ERG in human prostate cancers. Cancer Research. https://doi.org/10.1158/0008-5472.CAN-11-0876
THOMS, J. A. I., BIRGER, Y., FOSTER, S., KNEZEVIC, K., KIRSCHENBAUM, Y., CHANDRAKANTHAN, V., JONQUIERES, G., SPENSBERGER, D., WONG, J. W., ORAM, S. H., KINSTON, S. J., GRONER, Y., LOCK, R., MACKENZIE, K. L., GÖTTGENS, B., IZRAELI, S., & PIMANDA, J. E. 2011. ERG promotes T-acute lymphoblastic leukemia and is transcriptionally regulated in leukemic cells by a stem cell enhancer. Blood. https://doi.org/10.1182/blood-2010-12-317990

© 2022. Published by The Company of Biologists under the terms of the Creative Commons Attribution License (https://creativecommons.org/licenses/by/4.0/).

Resubmission

First decision letter

MS ID#: DEVELOP/2022/200528

MS TITLE: ERG activity is regulated by endothelial FAK coupling with TRIM25/USP9x in vascular patterning

AUTHORS: Gabriela D'Amico, Isabelle Fernandez, Jesus Gomez-Escudero, Hyojin Kim, Eleni Maniati, Muhammad Syahmi Azman, Faraz Mardakheh, Bryan Serrels, Alan Serrels, Maddy Parsons, Anthony Squire, Graeme Birdsey, Anna Randi, Rathi Gangeswaran, Louise Reynolds, Natalia Bodrug, Yaohe Wang, Jun Wang, Pascal Meier, and Kairbaan Hodivala-Dilke

Please accept our sincere apologise for the delay in getting the reviewers comments on your revised manuscript. This was due to an unfortunate IT error on the BenchPress system which failed to pick-up on a change to the email address of one of the reviewers - it only came to our attention last week.I have now received both referees reports on the above manuscript, and have reached a decision. The referees' comments are appended below, or you can access them online: please go to BenchPress and click on the 'Manuscripts with Decisions' queue in the Author Area.

The overall evaluation is very positive and we would like to publish a revised manuscript in Development. However Reviewer 1 has a very minor point that you can readily address with a very minor change to the main text.

Reviewer 1

Advance summary and potential significance to field

The article entitled “ERG activity is regulated by endothelial FAK coupling with TRIM25/USP9x in vascular patterning” investigates the mechanism by which FAK (or PTK2) regulates vascular patterning in the mouse retina in great depth. More specifically, the authors show that nuclear FAK in vascular endothelial cells interacts with the deubiquitinase USP9x and ubiquitin ligase TRIM25 to promote ERG and DLL4 expression levels. The study therefore proposes a completely novel mode of how the key angiogenic factors ERG, FAK and DLL4 functionally interact with each other by providing evidence for an FAK-mediated control of ERG phosphorylation and ubiquitination events to govern retinal angiogenesis. The report includes a remarkably high number of diverse state-of-the-art methods, including endothelial cell-specific inducible gene deletion mouse models, knock-in mouse models, LC-MS proteomic analyses retinal sprout analyses, endothelial competition assays, IP and interactome methods, Crispr/cas9 knockouts in endothelial cells as well as dynamic analyses of endothelial cell migration.

Comments for the author

The authors addressed my concerns in the revised manuscript. However, this referee strongly wishes these authors to describe in the MAIN TEXT that they used a Cre negative control, since this might be an issue of some experiments that the authors describe. This information shall not be hidden in the M&M section. Upon this addition, this referee recommends publication.

Reviewer 2

Advance summary and potential significance to field

This manuscripts provides interesting insights into how FAK regulates ERG during angiogenesis. Understanding the regulation of critical TF such as ERG is critical to fully understand how angiogenesis occurs.

Comments for the author

© 2022. Published by The Company of Biologists under the terms of the Creative Commons Attribution License (https://creativecommons.org/licenses/by/4.0/).

The authors have nicely addressed all of my comments. With the simplification of data, the manuscript has substantially been improved. It now nicely shows that FAK kinase activity and the phosphorylation at FAK-Y397 regulate ERG expression. In addition, they show that this occurs via the ubiquitin-mediated post-translational modification programme involving USP9x and TRIM25. There are no further comments.

Second revision

Author response to reviewers' comments

Reviewer 1:

Advance summary and potential significance to field

The article entitled “ERG activity is regulated by endothelial FAK coupling with TRIM25/USP9x in vascular patterning” investigates the mechanism by which FAK (or PTK2) regulates vascular patterning in the mouse retina in great depth. More specifically, the authors show that nuclear FAK in vascular endothelial cells interacts with the deubiquitinase USP9x and ubiquitin ligase TRIM25 to promote ERG and DLL4 expression levels. The study therefore proposes a completely novel mode of how the key angiogenic factors ERG, FAK and DLL4 functionally interact with each other by providing evidence for an FAK- mediated control of ERG phosphorylation and ubiquitination events to govern retinal angiogenesis. The report includes a remarkably high number of diverse state-of-the-art methods, including endothelial cell-specific inducible gene deletion mouse models, knock-in mouse models, LC-MS proteomic analyses, retinal sprout analyses, endothelial competition assays, IP and interactome methods, Crispr/cas9 knockouts in endothelial cells as well as dynamic analyses of endothelial cell migration.

Reviewer 1 Comments for the author

The authors addressed my concerns in the revised manuscript. However, this referee strongly wishes these authors to describe in the MAIN TEXT that they used a Cre negative control, since this might be an issue of some experiments that the authors describe. This information shall not be hidden in the M & M section. Upon this addition, this referee recommends publication.

We thank the reviewer for recommend the manuscript for publication after the addition of a short section of text. We have now incorporated clarification on the Cre-negatives to our revised MS: “Mice without Cre were used as control (Pdfgb-iCreERT-;FAK fl/fl)” in Line 136.

Reviewer 2:

Advance summary and potential significance to field

This manuscripts provides interesting insights into how FAK regulates ERG during angiogenesis. Understanding the regulation of critical TF such as ERG is critical to fully understand how angiogenesis occurs.

Reviewer 2 Comments for the author

The authors have nicely addressed all of my comments. With the simplification of data, the manuscript has substantially been improved. It now nicely shows that FAK kinase activity and the phosphorylation at FAK-Y397 regulate ERG expression. In addition, they show that this occurs via the ubiquitin-mediated post-translational modification programme involving USP9x and TRIM25. There are no further comments.

We thank the reviewer for highlighting that our work is of interest for publication.

© 2022. Published by The Company of Biologists under the terms of the Creative Commons Attribution License (https://creativecommons.org/licenses/by/4.0/).

Second decision letter

MS ID#: DEVELOP/2022/200528

MS TITLE: ERG activity is regulated by endothelial FAK coupling with TRIM25/USP9x in vascular patterning

AUTHORS: Gabriela D'Amico, Isabelle Fernandez, Jesus Gomez-Escudero, Hyojin Kim, Eleni Maniati, Muhammad Syahmi Azman, Faraz Mardakheh, Bryan Serrels, Alan Serrels, Maddy Parsons, Anthony Squire, Graeme Birdsey, Anna Randi, Rathi Gangeswaran, Louise Reynolds:, Natalia Bodrug, Yaohe Wang, Jun Wang, Pascal Meier, Alfonso Bolado-Carrancio, and Kairbaan Hodivala-Dilke
ARTICLE TYPE: Research Article

I am happy to tell you that your manuscript has been accepted for publication in Development, pending our standard ethics checks.

© 2022. Published by The Company of Biologists under the terms of the Creative Commons Attribution License (https://creativecommons.org/licenses/by/4.0/).
